# Risk factors for Hirschsprung disease-associated enterocolitis: a systematic review and meta-analysis

**DOI:** 10.1097/JS9.0000000000000473

**Published:** 2023-06-05

**Authors:** Xintao Zhang, Dong Sun, Qiongqian Xu, Han Liu, Yunfeng Li, Dongming Wang, Jian Wang, Qiangye Zhang, Peimin Hou, Weijing Mu, Chunling Jia, Aiwu Li

**Affiliations:** Departments ofa Pediatric surgery; b Gastroenterology; c Stomatology, Qilu Hospital of Shandong University, Jinan, China

**Keywords:** Enterocolitis, hirschsprungs disease, meta-analysis, risk factor, systematic review

## Abstract

**Background:**

The incidence of Hirschsprung disease (HSCR) is nearly 1/5000 and patients with HSCR are usually treated through surgical intervention. Hirschsprung disease-associated enterocolitis (HAEC) is a complication of HSCR with the highest morbidity and mortality in patients. The evidence on the risk factors for HAEC remains inconclusive to date.

**Methods:**

Four English databases and four Chinese databases were searched for relevant studies published until May 2022. The search retrieved 53 relevant studies. The retrieved studies were scored on the Newcastle–Ottawa Scale by three researchers. Revman 5.4 software was employed for data synthesis and analysis. Stata 16 software was employed for sensitivity analysis and bias analysis.

**Results:**

A total of 53 articles were retrieved from the database search, which included 10 012 cases of HSCR and 2310 cases of HAEC. The systematic analysis revealed anastomotic stenosis or fistula [*I* ^2^=66%, risk ratio (RR)=1.90, 95% CI 1.34–2.68, *P*<0.001], preoperative enterocolitis (*I* ^2^=55%, RR=2.07, 95% CI 1.71–2.51, *P*<0.001), preoperative malnutrition (*I* ^2^=0%, RR=1.96, 95% CI 1.52–2.53, *P*<0.001), preoperative respiratory infection or pneumonia (*I* ^2^=0%, RR=2.37, 95% CI 1.91–2.93, *P*<0.001), postoperative ileus (*I* ^2^=17%, RR=2.41, 95% CI 2.02–2.87, *P*<0.001), length of ganglionless segment greater than 30 cm (*I* ^2^=0%, RR=3.64, 95% CI 2.43–5.48, *P*<0.001), preoperative hypoproteinemia (*I* ^2^=0%, RR=1.91, 95% CI 1.44–2.54, *P*<0.001), and Down syndrome (*I* ^2^=29%, RR=1.65, 95% CI 1.32–2.07, *P*<0.001) as the risk factors for postoperative HAEC. Short-segment HSCR (*I* ^2^=46%, RR=0.62, 95% CI 0.54–0.71, *P*<0.001) and transanal operation (*I* ^2^=78%, RR=0.56, 95% CI 0.33–0.96, *P*=0.03) were revealed as the protective factors against postoperative HAEC. Preoperative malnutrition (*I* ^2^=35 ***%***, RR=5.33, 95% CI 2.68–10.60, *P*<0.001), preoperative hypoproteinemia (*I* ^2^=20%, RR=4.17, 95% CI 1.91–9.12, *P*<0.001), preoperative enterocolitis (*I* ^2^=45%, RR=3.51, 95% CI 2.54–4.84, *P*<0.001), and preoperative respiratory infection or pneumonia (*I* ^2^=0%, RR=7.20, 95% CI 4.00–12.94, *P*<0.001) were revealed as the risk factors for recurrent HAEC, while short-segment HSCR (*I* ^2^=0%, RR=0.40, 95% CI 0.21–0.76, *P*=0.005) was revealed as a protective factor against recurrent HAEC.

**Conclusion:**

The present review delineated the multiple risk factors for HAEC, which could assist in preventing the development of HAEC.

## Graphical abstract

**Figure:**
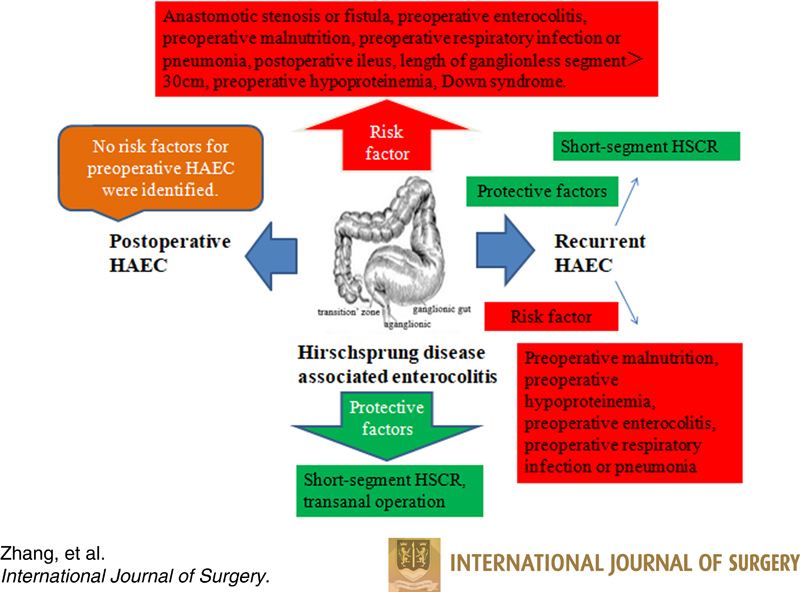

## Introduction

Highlights

- Hirschsprung disease-associated enterocolitis (HAEC) is the most serious and common complication of Hirschsprung disease and is the leading cause of death in Hirschsprung disease patients.
- Through the analyses we derived a series of risk factors for HAEC.
- The incidence of preoperative, postoperative, and recurrent HAEC was 23.31%, 23.10%, and 8.01%.
- This is the first and largest meta-analysis of HAEC-related risk factors.

Hirschsprung disease (HSCR), also referred to aganglionosis, is a congenital disorder of the intestinal tract^1^. The incidence of HSCR is ~1 in 5000 with a female-to-male ratio of ~1/4^2^. Its main pathogenesis is a developmental disorder of the enteric nervous system, which leads to intestinal motility disorders, and the main pathological manifestation is the absence of ganglion cells in the diseased intestine, which usually extend over specific distances^3^. HSCR is one of the first multi-gene genetic disorders to be identified, and so far, over 25 genes have been confirmed to be associated with HSCR^4–6^. However, not all cases of HSCR are reported to have genetic mutations, and in a large proportion of patients, HSCR presents with no associated mutations and is thought to be caused mainly due to environmental factors^2,7,8^.

Hirschsprung disease-associated enterocolitis (HAEC) is the most serious and common complication of HSCR^9^, and is also the leading cause of morbidity and mortality in patients with HSCR^10,11^. The clinical characteristics of HAEC include abdominal distention, diarrhoea, fever, and subsequent sepsis^12^. The incidence of HAEC ranges approximately from 17 to 50%, with the incidence of preoperative HAEC ranging from 5.7 to 50% and that of postoperative HAEC ranging from 2 to 25%. Recurrent HAEC occurs in 5.2–56% of HSCR patients^13^. These data demonstrate that HAEC is an important disease affecting the general well-being of patients with HSCR.

The pathogenesis of HAEC involves a complex interaction of dysfunction of the enteric nervous system, abnormal mucin secretion, inadequate immunoglobulin secretion, and imbalance of the intestinal microflora^14,15^. In addition, several controllable environmental factors are thought to be associated with the development of HAEC^16,17^. The genetic aspects^18^ such as Gfra1, microRNA-18a-5p, and OSMR^19–21^ are also being gradually revealed to be relevant to the development of HAEC.

An effective approach to prevent the development of HAEC remains to be discovered so far^22,23^. Identification of the risk factors for HAEC could assist in better prevention of the disease^13,24^. In this context, the present study aimed to systematically analyze the risk factors for HAEC.

## Materials and methods

The present systematic review and meta-analysis followed the recommendations of the Cochrane Handbook for Systematic Reviews of Interventions and was complied with the Preferred Reporting Items for Systematic Reviews and Meta-Analyses (PRISMA, Supplemental Digital Content 1, http://links.lww.com/JS9/A626, Supplemental Digital Content 2, http://links.lww.com/JS9/A627)^25,26^ statement and A MeaSurement Tool to Assess systematic Reviews 2 (AMSTAR2, Supplemental Digital Content 3, http://links.lww.com/JS9/A628)^27^.

### Inclusion and exclusion criteria

The inclusion criteria were as follows: (1) The duration of follow-up of the HAEC-related study was sufficiently long, at least over 2 years, to allow for a sufficient duration for the development of symptoms. (2) The pathological diagnosis of HSCR was established distinctly at the relevant hospital. (3) HSCR has a clear pathological diagnosis at the relevant hospital. (4) A minimum of one risk factor for HAEC was studied. (5) The study reported original data.

The exclusion criteria were: (1) Reviews, meta-analyses and articles without specific data. (2) Data replication in different studies. (3) Chinese non-core journals.

### Data sources

Three researchers searched PubMed, Web of Science, Embase, Cochrane Library, CNKI, Wanfang database, China Science and Technology Journal Database (VIP), and China Biomedical Literature Database (SinoMed) for relevant studies published until May 2022. The following terms were used for Hirschsprung disease: Hirschsprung disease, HSCR, HD, congenital megacolon, and aganglionosis. The keywords used for risk factors were: risk factor, related factors, influence factor, predictors, environmental factors, and every specific factor. The key search terms used for enterocolitis were: enterocolitis, coloenteritis, intestinal colitis, enteritis, enteronitis, esoenteritis, and HAEC. The detailed search strategy is presented in Appendix I, Supplemental Digital Content 4, http://links.lww.com/JS9/A629.

### Study selection

Three authors first selected all studies independently of each other and then met to review their choices to reach a consensus. Any inconsistencies were resolved through discussion to reach a common consensus.

### Data extraction

Data from all retrieved articles were extracted by three reviewers independently of each other. Any disagreements were resolved by a fourth reviewer. The data extracted from the articles included authors, the journal of publication, the year of publication, the study location, the type of study, the exposure cases, and the total cases.

### Risk/protective factor definition

In the present study, a risk factor was defined as follows: that characteristic, variable, or hazard preceding the outcome of interest, which, if present for a given individual, renders it further probable that this individual, rather than someone else selected from the general population, would develop a given disorder^28^. Similarly, protective factors were defined as those factors that led to reduced risk of morbidity. It was assumed that the risk/protective factors had occurred prior to HAEC.

The following are the definitions of the factors investigated in the present study:

#### Technical factors

Staged surgery was divided into two groups based on whether it was a one-stage procedure or a multiple-staged procedure. Intestinal preparation duration of less than 2 weeks was defined as less than two weeks of bowel preparation prior to surgery. Dietary control and dilatation were divided into two groups based on whether diet control and dilation were adopted or not.

#### Patient factors

Preoperative malnutrition and preoperative hypoproteinemia implied that the HSCR patients were diagnosed with malnutrition or hypoproteinemia prior to surgery. Preoperative respiratory infection or pneumonia was defined as the occurrence of respiratory infection or pneumonia in HSCR patients within 2 weeks prior to surgery. Operative age was defined as the age of the patient at the time of surgery, and because most of the retrieved studies had classified patients as older than 1 month or 1 year, this was used as a factor in the present study. The objective was to study the age factor as a continuous variable or to study the operative age less than 3 months; however, sufficient data to support this were not available in the literature.

#### Disease factors

Anastomotic stricture or fistula and postoperative ileus are the stenosis or fistula located at the anastomosis or ileus that occur after an HSCR surgery. Preoperative enterocolitis is the enterocolitis that occurs in HSCR patients prior to surgery. Pathological type refers to the pathological type of HSCR, and in the present study, patients were classified into short-segment type and other types. The length of the aganglionosis segment greater than 30 cm was selected as its length of the pathologically confirmed aganglionosis bowel over than 30 cm in HSCR patients.

### Quality assessment

The quality of the cohort and case-control studies was assessed using the Newcastle–Ottawa Scale scale^29,30^. The cohort studies assessed outcomes using the following three approaches: (1) representativeness of the exposure cohort. (2) comparability. (3) assessment of the time to outcome.

The quality of case-control studies was assessed by the following three approaches: (1) selection of the case and control groups; (2) comparability; (3) exposure^31^. The total score for the assessment was nine, and the inclusion criteria in the present meta-analysis were six or more.

### Statistical analysis

Analyses were initially completed by one author and subsequently reviewed by another author. RevMan 5.4 (Review manager) was employed to calculate the effect sizes and generate forest plots. The χ2 test was performed to analyze the statistical heterogeneity between studies, with *P* less than 0.05 indicating statistical significance. The Q tests were performed to quantitatively assess whether this heterogeneity would affect the results. According to the Cochrane review guidelines, fixed-effects models were used when the heterogeneity was small (*I* ^2^<50%); otherwise, a random-effects model was used for analysis. The original data on the previously published risk factors extracted from the retrieved studies were analyzed using dichotomous, relative risk (RR), 95% CI, and the calculations were performed using the Mantel Haenszel method (fixed or random model). Stata 16.0 (Stata Corp LP) was employed for sensitivity analysis and publication bias. Publication bias was determined using Egger’s test and funnel plot. *P* less than 0.05 was considered statistically significant.

## Results

### Search results and study characteristics

**Figure 1:**
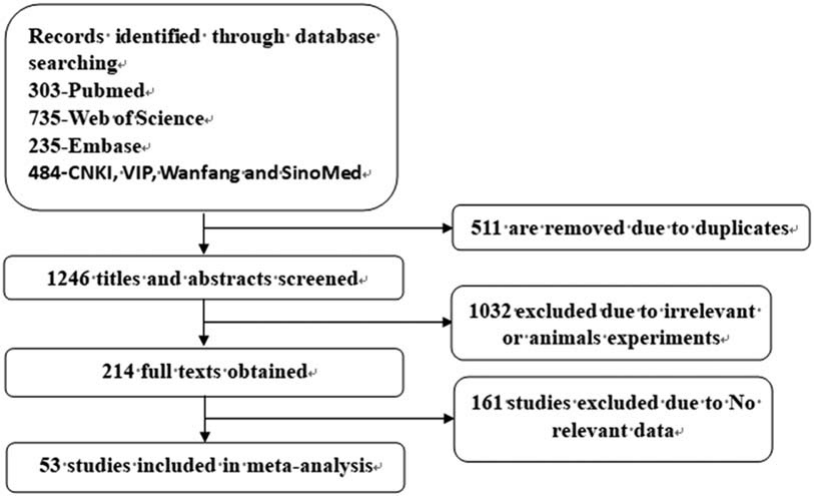
Flow chart of risk factors for HAEC study search and screening. HAEC, Hirschsprung disease-associated enterocolitis.

The search for the risk factors of HAEC retrieved 1757 studies (303 from PubMed, 735 from Web of Science, 235 from Embase, and 484 from CNKI, VIP, Wanfang, and Sinomed). Among the retrieved studies, 511 studies were excluded due to duplication, while 1032 studies were excluded due to irrelevant and non-core journals. The remaining 214 studies were read in detail, and eventually, 53 studies were retained. The literature screening process is illustrated in Figure 1, and the characteristics of these studies are listed in Table 1.

**Table 1:** Characteristics of the studies included in the meta-analysis.

Study ID	District	Country	Type of study	Journal	Case/total cases
Gunnarsdóttir ^32^	Lund	Sweden	Cohort study	European Journal of Pediatric Surgery	4/29
Nasr *et al*.^33^	Toronto	Canada	Cohort study	Journal of Pediatric Surgery	8/54
Yokoi *et al.* ^34^	Hyogo	Japan	Cohort study	Journal of Pediatric Surgery	27/89
Kubota *et al.* ^35^	Osaka	Japan	Cohort study	Journal of Pediatric Surgery	11/41
Pini Prato *et al.* ^36^	Genoa	Italy	Cohort study	Pediatric Surgery International	25/112
Pini Prato *et al.* ^37^	Genoa/Alessandria	Italy	Cohort study	Journal of Pediatric Surgery	87/324
Aliev^38^	Tashkent	Uzbekistan	Cohort study	European Journal of Molecular & Clinical Medicine	17/138
Tannuri^39^	São Paulo	Brazil	Cohort study	Journal of Pediatric Surgery	8/64
Kim *et al.* ^40^	Multicenter	American	Cohort study	Journal of Pediatric Surgery	105/281
Le-Nguyen *et al.* ^41^	Montreal	Canada	Cohort study	Journal of Pediatric Surgery	58/282
Morabito *et al.* ^42^	Manchester	United kingdom	Cohort study	Pediatric Surgery International	19/173
Zheng^43^	Xi an	China	Cohort study	Chinese Journal of Pediatric Surgery	8/65
Hackam^44^	Toronto	Canada	Cohort study	Journal of Pediatric Surgery	35/105
Hackam^45^	Toronto	Canada	Case-control study	Journal of Pediatric Surgery	33/105
Lu *et al.* ^46^	Nan jing	China	Cohort study	Journal of Pediatric Surgery	76/415
Shi^47^	Shang hai	China	Cohort study	Chinese Journal of Pediatric Surgery	16/103
Shen *et al.* ^48^	Shang hai	China	Cohort study	Journal of Pediatric Surgery	9/29
Hackam *et al.* ^49^	Pittsburgh	American	Cohort study	Pediatric Surgery International	19/173
Roorda *et al.* ^12^	Amsterdam	Netherlands	Cohort study	Journal of Pediatric Surgery	31/146
Mo^50^	Nanning	China	Case-control study	Master degree thesis of Guangxi Medical University	43/131
Teitelbaum^51^	Columbus, Ohio	American	Cohort study	Annals of surgery	19/80
Teitelbaum *et al.* ^52^	multicenter	American	Cohort study	Annals of surgery	55/181
Yulianda^53^	Yogyakarta	Indonesia	Case-control study	BMC Proceedings	11/61
Travassos^54^	Utrecht	Netherlands	Cohort study	Surgical Endoscopy	12/55
Gunadi^55^	Yogyakarta	Indonesia	Cohort study	BMC Research Notes	6/33
Gunadi *et al.* ^56^	Yogyakarta	Indonesia	Cohort study	BMC Pediatrics	14/83
Parahita^57^	Yogyakarta	Indonesia	Cohort study	Journal of Pediatric Surgery	15/100
Minford *et al.* ^58^	Liverpool	England	Cohort study	Journal of Pediatric Surgery	14/70
Sulkowski *et al.* ^59^	Ohio	American	Cohort study	Journal of Pediatric Surgery	430/1555
Li,^60^	Shang hai	China	Case-control study	Journal of Shanghai Jiao Tong University (Medical Science)	28/67
Pruitt *et al.* ^61^	national	American	Cohort study	Journal of Pediatric Surgery	265/2030
Santos^62^	Johnson City	American	Cohort study	Journal of Pediatric Surgery	11/65
Menezes^63^	Dublin	Ireland	Cohort study	Pediatric Surgery International	74/259
Taylor *et al.* ^64^	Salt Lake City	American	Cohort study	European Journal of Pediatric Surgery	140/299
Nakamura *et al.* ^65^	Tokyo	Japan	Cohort study	Pediatric Surgery International	7/64
Kwendakwema *et al.* ^66^	Salt Lake City	American	Cohort study	Journal of Pediatric Surgery	79/207
Ishikawa *et al.* ^67^	Osaka	Japan	Cohort study	Pediatric Surgery International volume	12/49
Aworanti *et al.* ^68^	Dublin	Ireland	Cohort study	European Journal of Pediatric Surgery	19/73
Liang^69^	Xin Jiang	China	Cohort study	Master degree thesis of Xinjiang Medical University	18/60
Romero *et al.* ^70^	Manheim	Germany	Cohort study	Langenbeck’s Archives of Surgery	5/53
Dong^71^	Guangzhou	China	Cohort study	Journal of Pediatric Surgery	61/133
Surana^72^	Dublin	Ireland	Cohort study	Pediatric Surgery International	41/135
Singh *et al.* ^73^	Ontario	Canada	Cohort study	Journal of Pediatric Surgery	10/77
Agarwala^74^	New Delhi	India	Cohort study	Department of Pediatric Surgery	2/50
Tang^75^	Wuhan	China	Cohort study	Chinese Journal of Pediatric Surgery	34/141
Zhang *et al.* ^76^	Shengyang	China	Cohort study	Journal of Pediatric Surgery	3/58
Chia *et al.* ^77^	Taiwan	China	Cohort study	Pediatrics & Neonatology	169/629
Kim^78^	Daegu	Korea	Cohort study	Journal of the Korean Surgical Society	6/36
Giuliani *et al.* ^79^	Los Angeles	American	Cohort study	Journal of Laparoendoscopic & Advanced Surgical	10/140
Sakurai^80^	Sendai	Japan	Case-control study	Pediatric Surgery International volume	12/66
Saleh *et al.* ^81^	Riyadh	Saudi Arabia	Cohort study	Pediatric Surgery International	3/38
Huang *et al.* ^82^	Shenyang	China	Cohort study	Journal of Gastrointestinal Surgery	52/181
Wang^83^	Nanning	China	Cohort study	Master degree thesis of Guangxi Medical University	73/163

### Quality assessment of the studies

Among the included studies, the Newcastle–Ottawa Scale score was eight for six studies, seven for twenty-eight studies, and six for the remaining studies. Among the studies included in the present meta-analysis, forty-eight were cohort studies, and five were case-control studies (forty-seven were in English, and six were in Chinese). Further details of the quality assessment are provided in Table 2 and Table 3.

**Table 2:** Quality evaluation of the case-control studies included in meta-analysis.

	Selection				Comparability		Outcome		
Study ID	Is the case definition adequate	Representativeness of the cases	Selection of Controls	Definition of Controls	Comparability of cases and controls on the basis of the design or analysis	Ascertainment of exposure	Same method of ascertainment for cases and controls	Non-response rate	Quality assessment score
Hackam^45^	★		★	★	★	★	★	★	7
Mo^50^	★		★	★	★		★	★	7
Yulianda^53^	★		★		★	★	★	★	6
Li^60^	★		★	★	★	★	★	★	7
Sakurai^80^	★		★	★	★	★	★	★	7

**Table 3:** Quality evaluation of the cohort studies included in meta-analysis.

	Selection				Comparability	Exposure			
Study ID	Representativeness of exposure cohort	Selection of non-exposed cohort	Ascertainment of exposure cohort	Demonstration that outcome of interest was not present at start of study	Comparability of exposed and non-exposed cohorts	Method for determination of results	Was the follow-up time long enough	Completeness of follow-up cohort	Quality assessment score
Gunnarsdóttir^32^		★	★	★	★	★		★	6
Nasr *et al.* ^33^		★	★	★	★	★	★	★	7
Yokoi *et al.* ^34^		★	★	★	★	★	★	★	7
Kubota *et al.* ^35^	★	★	★	★	★			★	6
Pini Prato *et al.* ^36^	★	★	★	★	★	★		★	7
Pini Prato *et al.* ^37^	★	★	★	★	★★	★		★	8
Aliev^38^		★	★	★	★	★		★	6
Tannuri^39^		★	★	★	★	★	★	★	7
Kim *et al.* ^40^		★	★	★	★	★	★		6
Nguyen *et al.* ^41^		★	★	★	★	★		★	6
Morabito *et al.* ^42^		★	★	★	★	★	★	★	7
Zheng^43^	★	★	★	★	★	★		★	7
Hackam^44^		★	★	★	★	★	★	★	7
Lu *et al.* ^46^		★	★	★	★	★	★	★	7
Shi^47^		★	★	★	★	★		★	6
Shen *et al.* ^48^		★	★	★	★	★		★	6
Hackam *et al.* ^49^		★	★	★	★		★	★	6
Roorda *et al.* ^12^	★	★	★	★	★	★	★	★	8
Teitelbaum^51^	★	★	★	★	★	★	★	★	8
Teitelbaum *et al.* ^52^		★	★	★	★	★	★	★	7
Travassos^54^		★	★	★	★	★	★	★	7
Gunadi^55^		★	★	★	★	★		★	6
Gunadi *et al.* ^56^		★	★	★	★	★	★	★	7
Parahita^57^		★	★	★	★	★	★	★	7
Minford *et al.* ^58^		★	★	★	★	★		★	6
Sulkowski *et al.* ^59^	★	★	★	★	★★		★	★	8
Pruitt *et al.* ^61^	★	★	★	★	★★		★	★	8
Santos^62^		★	★	★	★	★	★	★	7
Menezes^63^	★	★	★	★	★			★	6
Taylor *et al.* ^64^	★	★	★	★	★	★		★	7
Nakamura *et al.* ^65^		★	★	★	★	★		★	6
Kwendakwema *et al.* ^66^	★	★	★	★	★	★		★	7
Ishikawa *et al.* ^67^	★	★	★	★	★	★		★	6
Aworanti *et al.* ^68^	★	★	★	★	★		★	★	7
Liang^69^	★	★	★	★	★	★		★	7
Romero *et al.* ^70^		★	★	★	★	★	★	★	7
Dong^71^		★	★	★	★	★	★	★	7
Surana^72^		★	★	★	★	★	★	★	7
Singh *et al.* ^73^		★	★	★	★		★	★	6
Agarwala^74^		★	★	★	★		★	★	6
Tang^75^	★	★	★	★	★	★		★	7
Zhang *et al.* ^76^		★	★	★	★	★		★	6
Chia *et al.* ^77^	★	★	★	★	★★	★		★	8
Kim^78^		★	★	★	★		★	★	6
Giuliani *et al.* ^79^	★	★	★	★	★		★	★	7
Saleh *et al.* ^81^		★	★	★	★	★	★	★	7
Huang *et al.* ^82^		★	★	★	★	★	★	★	7
Wang^83^		★	★	★	★	★		★	6

### Quantitative synthesis

#### Incidence

In total, the 53 studies on HAEC contained 2310 cases of HAEC and 10 012 cases of HSCR^12,32–83^. The proportion of postoperative incidence of HAEC cases in these studies (HAEC cases>10) ranged from 10.98 to 46.82% of the HSCR patients, with a total incidence of 23.10%.The incidence of preoperative HAEC ranged from 10.77 to 46.15%, with a total incidence of 21.31%.The incidence of recurrent HAEC ranged from 6.80 to 18.05%, with a total incidence of 8.01%.

#### Risk factors for postoperative HAEC

##### Patient factors

###### Sex.

**Figure 2:**
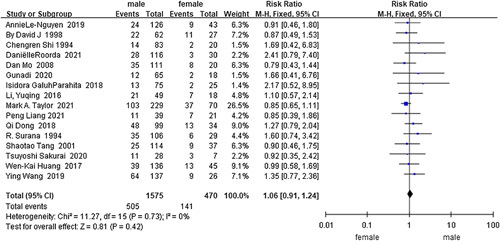
Forest plot of sex for postoperative HAEC. HAEC, Hirschsprung disease-associated enterocolitis.

Sex^12,41,45,47,50,56,57,60,64,69,71,72,75,80,82,83^ (*I* ^2^=0%, RR=1.06, 95% CI 0.91–1.24, *P*=0.42) (Fig. 2) was not a risk factor for HAEC, as determined based on the analysis of the data from 4 case-control studies and 12 cohort studies with 2045 HSCR and 646 HAEC cases. The analysis of the case-control studies (*I* ^2^=0%, RR=0.90, 95% CI 0.65–1.26, *P*=0.55) or cohort studies(*I* ^2^=0%, RR=1.11, 95% CI 0.93–1.31, *P*=0.25) alone did not change the above finding. Detailed results of the analyses conducted for all factors separately for the case-control study or the cohort study, along with the forest plots, are presented in Appendix II, Supplemental Digital Content 4, http://links.lww.com/JS9/A629.

###### Operative age.

**Figure 3:**
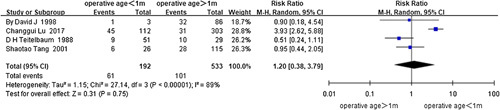
Forest plot of age of surgery more than 1 month for postoperative HAEC. HAEC, Hirschsprung disease-associated enterocolitis.

**Figure 4:**
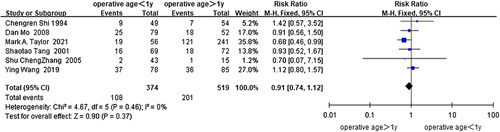
Forest plot of age of surgery more than 1 year for postoperative HAEC. HAEC, Hirschsprung disease-associated enterocolitis.

No statistical significance was revealed for the age of surgery more than 1 month^45,46,51,75^ (*I* ^2^=89%, RR=1.20, 95% CI 0.38–3.79, *P*=0.75) (Fig. 3) and the age of surgery more than 1 year^47,50,64,75,76,83^ (*I* ^2^=0%, RR=0.91, 95% CI 0.74–1.12, *P*=0.37) (Fig. 4) in the analysis of the data from three cohort studies and one case-control study, and five cohort studies and one case-control study, respectively. Analysis of the case-control studies or cohort studies alone did not change the above results.

###### Preoperative malnutrition.

**Figure 5:**
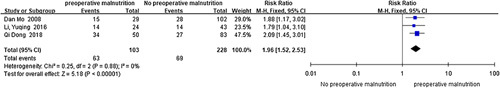
Forest plot of preoperative malnutrition for postoperative HAEC. HAEC, Hirschsprung disease-associated enterocolitis.

Preoperative malnutrition (*I* ^2^=0%, RR=1.96, 95% CI 1.52–2.53, *P*<0.001) ( **Fig. 5** ) was revealed as a risk factor for HAEC in the analysis of the data from one cohort study and two case-control studies with 331 HSCR and 132 HAEC cases. The analysis of case-control studies^60,84^ [*I* ^2^=0%, odds ratio (OR)=2.86, 95% CI 1.48–5.51, *P*=0.002] or cohort studies^71^ (*I* ^2^=0%, RR=4.41, 95% CI 2.08–9.34, *P*<0.001) alone did not change the above finding.

###### Preoperative respiratory infection or pneumonia.

**Figure 6:**
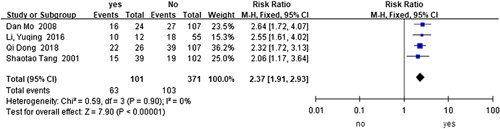
Forest plot of preoperative hypoproteinemia for postoperative HAEC. HAEC, Hirschsprung disease-associated enterocolitis.

Preoperative respiratory infection or pneumonia^50,60,71,75^ (*I* ^2^=0%, RR=2.37, 95% CI 1.91–2.93, *P*<0.001) (Fig. 6) was revealed as a risk factor for HAEC in the analysis of the data from two cohort studies and two case-control studies with 472 HSCR and 166 HAEC cases. The analysis of case-control studies^50,60^ (*I* ^2^=0%, OR=7.00, 95% CI 3.09–15.84, *P*<0.001) or cohort studies^71,75^ (*I* ^2^=0%, RR=2.22, 95% CI 1.66–2.96, *P*<0.001) alone did not change the above finding.

###### Preoperative hypoproteinemia.

**Figure 7:**
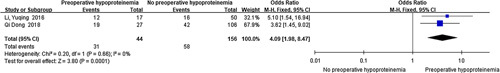
Forest plot of preoperative respiratory infection or pneumonia for postoperative HAEC. HAEC, Hirschsprung disease-associated enterocolitis.

The analysis of data from two studies revealed that preoperative hypoproteinemia^60,71^ (*I* ^2^=0%, RR=1.91, 95% CI 1.44–2.54, *P*<0.001) (Fig. 7) was a risk factor for postoperative HAEC.

##### *Technical factors*

###### Surgical methods and access.

**Figure 8:**
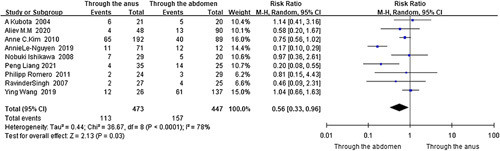
Forest plot of transanal operation for postoperative HAEC. HAEC, Hirschsprung disease-associated enterocolitis.

The analysis of data for 253 HAEC and 782 HSCR from eight cohort studies and one case-control study revealed that compared with transabdominal, transanal^35,38,40,41,67,69,70,73,83^ (*I* ^2^=78%, RR=0.56, 95% CI 0.33–0.96, *P*=0.03) (Fig. 8) is a protective factor for HAEC. Moreover, comparisons of Soave and Duhamel^45,48,56,57,63–65,71,72,78,80,82^ (nine cohort studies and two case-control studies, *I* ^2^=37%, RR=0.95, 95% CI 0.69–1.30, *P*=0.75), Swenson and Soave^38,45,63,64,72,82^ (five cohort studies and one case-control study, *I* ^2^=88%, RR=1.31, 95% CI 0.70–2.48, *P*=0.40), Duhamel and transanal endorectal pull-through (TEPT)^32,39,50,55,63,79^ (six cohort studies, *I* ^2^=0%, RR=0.93, 95% CI 0.59–1.45, *P*=0.74), Swenson and Duhamel^45,63,64,72^ (three cohort studies and one case-control study, *I* ^2^=0%, RR=0.95, 95% CI 0.67–1.33, *P*=0.75), laparotomy and laparoscope^12,34,41,54,69,71,79,82,83^ (nine cohort studies, *I* ^2^=23%, RR=1.14, 95% CI 0.87–1.49, *P*=0.35) did not reveal statistically significant differences.

###### Other technical factors.

The analysis of the data from seven cohort studies and one case-control study revealed no statistical significance for staged surgery^43,44,52,58,59,62,75^ (*I* ^2^=75%, RR=1.19, 95% CI 0.75–1.90, *P*=0.46). The data from two cohort studies were analyzed, and no statistical significance was revealed for the intestinal preparation time of less than 2 week^69,82^ (*I* ^2^=88%, RR=1.31, 95% CI 0.38–4.48, *P*=0.67) and dietary control^69,82^ (*I* ^2^=77%, RR=0.42, 95% CI 0.14–1.27, *P*=0.12). No statistical significance was revealed for anal dilatation^68,69,82,83^ (*I* ^2^=0%, RR=1.21, 95% CI 0.92–1.60, *P*=0.17) in the analysis of data from four cohort studies.

##### *Disease factors*

###### Anastomotic stricture or fistula.

**Figure 9:**
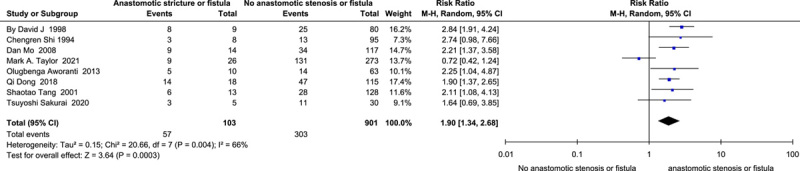
Forest plot of anastomotic stenosis or fistula for postoperative HAEC. HAEC, Hirschsprung disease-associated enterocolitis.

The analysis of the data from five cohort studies and three case-control studies containing 1004 HSCR and 360 HAEC cases revealed that anastomotic stenosis or fistula^45,47,50,64,68,71,75,80^ (*I* ^2^=66%, RR=1.90, 95% CI 1.34–2.68, *P*<0.001) (Fig. 9) was a risk factor for HAEC. The analysis of the case-control studies^45,50,80^ (*I* ^2^=0%, OR=5.56, 95% CI 2.33–13.28, *P*<0.001) or cohort studies^47,64,68,71,75^ (*I* ^2^=70%, RR=1.69, 95% CI 1.02–2.79, *P*=0.04) separately did not change the above finding.

###### Preoperative enterocolitis.

**Figure 10:**
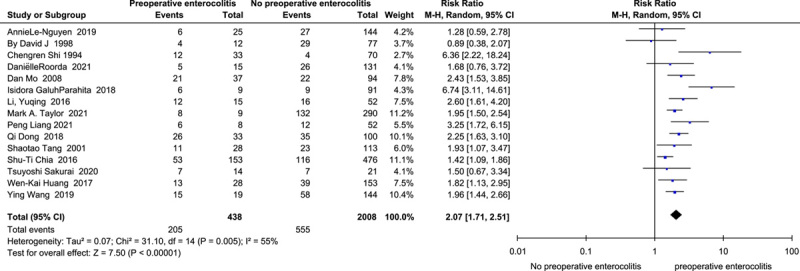
Forest plot of preoperative enterocolitis for postoperative HAEC. HAEC, Hirschsprung disease-associated enterocolitis.

Preoperative enterocolitis^12,41,45,47,50,57,60,64,69,71,75,77,80,82,83^ (*I* ^2^=55%, RR=2.07, 95% CI 1.71–2.51, *P*<0.001) (Fig. 10) was revealed as a risk factor for HAEC in the analysis of the data of 2446 HSCR and760 HAEC cases from eleven cohort studies and four case-control studies. The analysis of the case-control studies^45,50,60,80^ (*I* ^2^=59%, OR=2.89, 95% CI 1.14–7.33, *P*=0.03) or cohort studies^12,41,47,57,64,69,71,75,77,82,83^ (*I* ^2^=60%, RR=2.12, 95% CI 1.70–2.65, *P*<0.001) alone did not change the above finding.

###### Pathological type.

**Figure 11:**
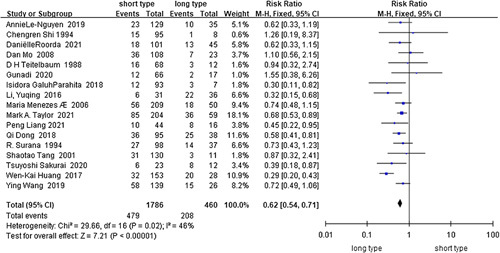
Forest plot of short-segment HSCR for postoperative HAEC. HAEC, Hirschsprung disease-associated enterocolitis; HSCR, Hirschsprung disease.

Short-segment HSCR^12,41,47,50,51,56,57,60,63,64,69,71,72,75,80,82,83^ (*I* ^2^=46%, RR=0.62, 95% CI 0.54–0.71, *P*<0.001) (Fig. 11) was revealed as a protective factor for HAEC in the analysis of the data on 2246 HSCR and 687 HAEC cases from fourteen cohort studies and three case-control studies. The analysis of the case-control studies^50,60,80^ (*I* ^2^=70%, RR=0.55, 95% CI 0.36–0.83, *P*=0.004) or cohort studies^12,41,47,51,56,57,63,64,69,71,72,75,82,83^ (*I* ^2^=43%, RR=0.63, 95% CI 0.55–0.72, *P*<0.001) alone did not change the above finding.

###### Postoperative ileus.

**Figure 12:**
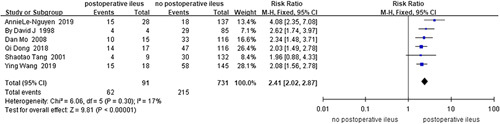
Forest plot of postoperative ileus for postoperative HAEC. HAEC, Hirschsprung disease-associated enterocolitis.

Postoperative ileus^41,45,50,71,75,83^ (*I* ^2^=17%, RR=2.41, 95% CI 2.02–2.87, *P*<0.001) (Fig. 12) was revealed as a risk factor for HAEC in the analysis of the data on 822 HSCR and 277 HAEC cases from four cohort studies and two case-control studies. The analysis of the case-control studies^45,50^ (*I* ^2^=0%, OR=6.42, 95% CI 2.25–18.34, *P*<0.001) or cohort studies^41,71,75,83^ (*I* ^2^=49%, RR=2.40, 95% CI 1.96–2.95, *P*<0.001) alone did not change the above finding.

###### Down syndrome.

**Figure 13:**
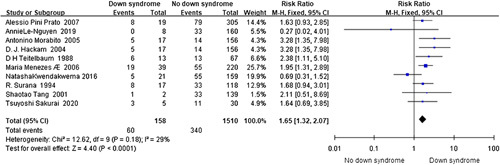
Forest plot of Down syndrome for postoperative HAEC. HAEC, Hirschsprung disease-associated enterocolitis.

Down syndrome^37,41,42,49,51,63,66,72,75,80^ (*I* ^2^=29%, RR=1.65, 95% CI 1.32–2.07, *P*<0.001) (Fig. 13) was revealed a risk factor for HAEC in the analysis of the data on 158 Down syndrome, 400 HAEC, and 1668 HSCR cases from nine cohort studies and a case-control study. This finding did not change when the cohort studies^37,41,42,49,51,63,66,72,75^ (*I* ^2^=37%, RR=1.65, 95% CI 1.31–2.08, *P*<0.001) were analyzed individually.

###### Length of the ganglionless segment.

**Figure 14:**
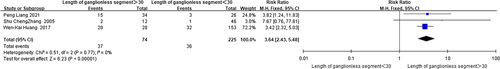
Forest plot of length of ganglionless segment for postoperative HAEC. HAEC, Hirschsprung disease-associated enterocolitis.

The analysis of the data from three studies and two studies revealed that the length of the ganglionless segment greater than 30 cm^69,76,82^ (*I* ^2^=0%, RR=3.64, 95% CI 2.43–5.48, *P*<0.001) (Fig. 14) was a risk factor for postoperative HAEC.

#### Risk factors for preoperative HAEC

The analyses of the extracted data revealed that sex^41,53^ (two studies, *I* ^2^=24%, RR=1.30, 95% CI 0.61–2.80, *P*=0.49), type of pathology^41,53^ (two studies, *I* ^2^=0%, RR=0.66, 95% CI 0.33–1.33, *P*=0.24) and age of surgery more than 1 month^49,53,77^ (three studies, *I* ^2^=92%, RR=0.95, 95% CI 0.18–4.96, *P*=0.95) were not statistically associated with preoperative HAEC.

#### Risk factors for recurrent HAEC

**Figure 15:**
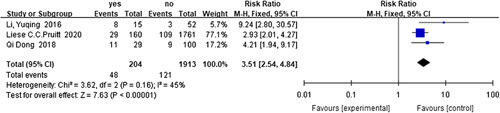
Forest plot of preoperative enterocolitis for recurrent HAEC. HAEC, Hirschsprung disease-associated enterocolitis.

**Figure 16:**
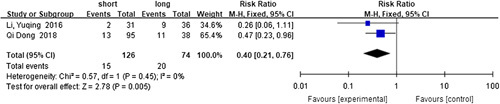
Forest plot of short-segment HSCR for recurrent HAEC. HAEC, Hirschsprung disease-associated enterocolitis; HSCR, Hirschsprung disease.

**Figure 17:**
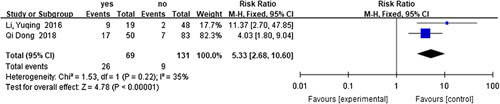
Forest plot of preoperative malnutrition for recurrent HAEC. HAEC, Hirschsprung disease-associated enterocolitis.

**Figure 18:**
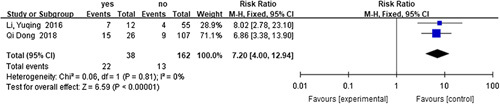
Forest plot of preoperative respiratory tract infection or pneumonia for recurrent HAEC. HAEC, Hirschsprung disease-associated enterocolitis.

**Figure 19:**
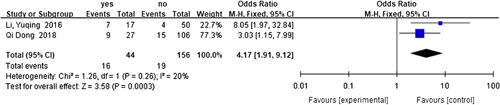
Forest plot of preoperative hypoproteinemia for recurrent HAEC. HAEC, Hirschsprung disease-associated enterocolitis.

The analysis of the data on 2230 HSCR patients, including 173 recurrent cases, from three studies, revealed preoperative EC^60,61,71^ (*I* ^2^=45%, RR=3.51, 95% CI 2.54–4.84, *P*<0.001) (Fig. 15) as a risk factor for recurrent HAEC, while sex^60,61,71^ (*I* ^2^=0%, RR=1.04, 95% CI 0.74–1.46, *P*=0.83) was revealed not to have a statistically significant association with this disease. The analysis of the data from two studies revealed short-segment HSCR^60,71^ (*I* ^2^=0%, RR=0.40, 95% CI 0.21–0.76, *P*=0.005) (Fig. 16) as a protective factor, while preoperative malnutrition^60,71^ (*I* ^2^=35%, RR=5.33, 95% CI 2.68–10.60, *P*<0.001) (Fig. 17), preoperative respiratory tract infection or pneumonia^60,71^ (*I* ^2^=0%, RR=7.20, 95% CI 4.00–12.94, *P*<0.001) (Fig. 18), and hypoproteinemia^60,71^ (*I* ^2^=20%, RR=4.17, 95% CI 1.91–9.12, *P*<0.001) (Fig. 19) were revealed as the risk factors for recurrent HAEC. The details of the forest plots for the non-statistically significant factors are provided in Appendix III, Supplemental Digital Content 4, http://links.lww.com/JS9/A629.

### Sensitivity analysis and publication bias

Sensitivity remained largely stable after the removal of any of the studies, with little influence on the outcomes. No significant asymmetry was detected in the funnel plot. Moreover, no evident publication bias was detected using Egger’s test (*P*>0.05). The associated funnel plot and Egger’s test results are presented in Appendix IV, Supplemental Digital Content 4, http://links.lww.com/JS9/A629.

## Discussion

In the present systematic review and meta-analysis, the factors associated with HAEC were explored, and 31 factors associated with HAEC were analyzed (Table 4). The results revealed eight risk factors and two protective factors significantly associated with postoperative HAEC. In addition, four risk factors and one protective factor associated with recurrent HAEC were revealed. No risk factors or protective factors were revealed for preoperative HAEC.

**Table 4:** Risk factors of postoperative HAEC, preoperative HAEC and recurrent HAEC.

Factors studied	Exposure/case	Non-exposed/ control	*I* ^2^, (%)	*P* for heterogeneity	Model	RR/OR	95% CI	*P*
Risk factors for postoperative HAEC.								
Anal dilatation^68,69,82,83^	285	192	0	0.450	Fixed	1.21	0.92–1.60	0.170
Anastomotic stricture or fistula^45,47,50,64,68,71,75,80^	103	901	66	0.004	Random	1.63	1.34–2.00	**<0.001**
Preoperative enterocolitis^12,41,45,47,50,57,60,64,69,71,75,77,80,82,83^	438	2008	55	0.005	Random	2.07	1.71–2.51	**<0.001**
Sex^12,41,45,47,50,56,57,60,64,69,71,72,75,80,82,83^	1575	470	0	0.730	Fixed	1.06	0.91–1.24	0.420
Pathological type (Short-segment HSCR)^12,41,47,50,51,56,57,60,63,64,69,71,72,75,80,82,83^	1786	470	46	0.020	Fixed	0.62	0.54–0.71	**<0.001**
Preoperative malnutrition^50,60,71^	103	228	0	0.700	Fixed	3.46	2.12–5.66	**<0.001**
Preoperative respiratory infection or pneumonia^50,60,71,75^	101	371	0	0.900	Fixed	2.37	1.91–2.93	**<0.001**
Postoperative ileus^41,45,50,71,75,83^	91	731	17	0.300	Fixed	2.41	2.02–2.87	**<0.001**
Operative age>1 mo^45,46,51,75^	192	533	89	<0.001	Random	1.20	0.38–3.79	0.750
Operative age>1 y^47,50,64,75,76,83^	374	519	0	0.460	Fixed	0.91	0.74–1.12	0.370
Staging operation (one-stage operation)^43,44,52,58,59,62,75^	1479	703	75	<0.001	Random	1.19	0.75–1.90	0.460
Intestinal preparation time>2 wk^69,82^	144	97	88	0.004	Random	1.31	0.38–4.48	0.670
Dietary control^69,82^	155	86	77	0.040	Random	0.42	0.14–1.27	0.120
Length of ganglionless segment>30cm^69,76,82^	74	225	0	0.770	Fixed	3.64	2.43–5.48	**<0.001**
Preoperative hypoproteinemia^60,71^	44	156	0	0.480	Fixed	1.91	1.44–2.54	**<0.001**
Down syndrome^37,41,42,49,51,63,66,72,75,80^	158	1510	29	0.180	Fixed	1.65	1.32–2.07	**<0.001**
Comparison of Soave and Duhamel^45,48,56,57,63–65,71,72,78,80,82^	647	369	36	0.110	Fixed	0.97	0.78–1.20	0.750
Comparison of Swenson and Soave^38,45,63,64,72,82^	258	592	88	<0.001	Random	1.31	0.70–2.48	0.400
Comparison of Duhamel and TEPT^32,39,50,55,63,79^	222	184	0	0.450	Fixed	0.93	0.59–1.45	0.740
Comparison of Swenson and Duhamel^45,63,64,72^	224	136	0	0.920	Fixed	0.95	0.67–1.33	0.750
Comparison of laparotomy and laparoscope^12,34,41,54,69,71,79,82,83^	393	287	23	0.240	Fixed	1.14	0.87–1.49	0.350
Comparison of transanal and transabdominal^35,38,40,41,67,69,70,73,83^	473	447	78	<0.001	Random	0.56	0.33–0.96	**0.030**
Risk factors for preoperative HAEC.								
Age of surgery >1 mo^49,53,77^	232	589	92	<0.001	Random	0.95	0.18–4.96	0.950
Sex^41,53^	176	56	24	0.250	Fixed	1.30	0.61–2.80	0.490
Pathological type (Short-segment HSCR)^41,53^	184	42	0	0.430	Fixed	0.66	0.33–1.33	0.240
Risk factors for recurrent HAEC.								
Sex^60,61,71^	1706	524	0	0.870	Fixed	1.04	0.74–1.46	0.830
Pathological type (Short-segment HSCR)^60,71^	126	74	0	0.450	Fixed	0.40	0.21–0.76	**0.005**
Preoperative malnutrition^60,71^	69	131	35	0.220	Fixed	5.33	2.68–10.60	**<0.001**
Preoperative EC^60,61,71^	204	1913	45	0.160	Fixed	3.51	2.54–4.84	**<0.001**
Preoperative respiratory infection or pneumonia^60,71^	38	162	0	0.810	Fixed	7.20	4.00–12.94	**<0.001**
Preoperative hypoproteinemia^60,71^	44	156	20	0.260	Fixed	4.17	1.91–9.12	**<0.001**

Statistically significant values are in bold.

HAEC, Hirschsprung disease-associated enterocolitis; EC, enterocolitis; HSCR, Hirschsprung disease; OR, odds ratio; RR, risk ratio; TEPT, transanal endorectal pull-through.

The debate on the risk factors for HAEC continues to date. Roorda *et al*.^12^ reported that preoperative EC, short-segment HSCR, and surgical methods were not associated with the development of HAEC, while the age of the patient during surgery was associated with HAEC. In contrast, Gao *et al.* ^85^ and certain other scholars^77,80,86^ have demonstrated the association of surgical methods, short-segment HSCR, and preoperative EC with the development of HAEC. In certain other studies, Soave was reported as a protective factor for HAEC^57^ while some do not^87^. These factors were analyzed comprehensively in the present study, and a number of factors associated with HAEC were identified. This is expected to have a positive impact on the relevant clinical work. When a patient presents with multiple risk factors for HAEC, the possibility of the development of HAEC must be considered. When a child suffers preoperative malnutrition, preoperative respiratory infection or pneumonia, and preoperative hypoproteinemia, it is appropriate to postpone the surgery. After correcting these symptoms without delaying the disease, the surgery should then be performed.

In addition to the 31 factors analyzed in the present meta-analysis, a number of other factors are thought to be potentially associated with HAEC. For instance, oblique anastomosis^88^, distance of the ganglion site from the incision margin^89^, low IgA levels, low birth weight^82^, birth Apgar score^12^, and milk allergy^90^ could be the risk factors for HAEC. HSCR complications are reportedly associated with other congenital malformations, maternal age older than 35 years, preterm birth^53^. However, due to insufficient data available in the literature, further analysis could not be conducted in the present study.

In comparison with other similar studies, Mao *et al.* ^91^ concluded that the incidence of HAEC was lower in Duhamel compared with TEPT. In contrast, different conclusions were reached in the present study due to the inclusion of greater literature for comparison. The results of the present study revealed no difference in the incidence of HAEC between the two surgical procedures. A meta-analysis by Ruttenstock *et al.* ^92^ revealed that TEPT is a safe and less-invasive procedure with a low incidence of postoperative HAEC. This was consistent with the findings of the present study. In addition, three studies^11,22,93^ on the association of probiotics with HAEC, which were conducted by Nakamura *et al*., presented a consistent conclusion that probiotics were not statistically correlated with HAEC. Since no new studies have been published after the literature search process was completed for the present studying, this factor was not updated and included as such in the results of the present study.

The major strength of the present study is that it is the first meta-analysis of the risk factors for HAEC. Moreover, it is the largest study conducted on HAEC to date. Multiple factors for preoperative, postoperative, and recurrent HAEC were analyzed separately. However, as with all research, the present study also had certain limitations. The diagnostic criteria were not the same among the different studies, although consistent clinical symptoms of HAEC were used in all diagnostic methods. In addition, only Chinese and English databases were searched, and the data obtained from the search may not be comprehensive. For certain factors, such as dietary control, intestinal preparation time, only a few studies were included, which resulted in a small sample size for such studies.

## Conclusion

The present review discusses the meta-analysis of 53 studies, which revealed eight risk factors and two protective factors for postoperative HAEC. Four risk factors and one protective factor for recurrent HAEC were obtained. No risk factors for preoperative HAEC were identified.

## Ethical approval

This study is a meta-analysis and ethics statement is not applicable.

## Funding

This research was funded by the National Natural Science Foundation of China (Project nos. 81873846 and 82071682).

## Author contribution

X.Z.: conceptualization, data curation, formal analysis, investigation, methodology, project administration, validation, writing—original draft and visualization. D.S., MD: data curation, formal analysis, methodology and visualization. Q.X.: data curation, formal analysis, methodology and visualization. H.L.: methodology, writing—review and editing. Y.L.: formal analysis, writing—review and editing. D.W.: formal analysis and investigation. J.W.: formal analysis, investigationand and methodology. Q.Z.: formal analysis and investigation. P.H.: formal analysis and methodology. W.M.: investigation and methodology. C.J.: conceptualization, formal analysis, methodology, project administration, resources, supervision and writing—review and editing. A.L.: conceptualization, formal analysis, funding acquisition, methodology, project administration, resources, supervision and writing—review and editing. All authors read and approved the final manuscript.

## Conflicts of interest disclosure

None.

## Research registration unique identifying number (UIN)

1. Name of the registry: Prospero.
2. Unique Identifying number or registration ID: CRD42022353241.
3. Hyperlink to your specific registration (must be publicly accessible and will be checked): https://www.crd.york.ac.uk/PROSPERO/display_record.php?RecordID =353241

## Guarantor

Aiwu Li.

## Data statement

This is a meta-analysis article, data availability is not applicable, please contact the corresponding author if some data needed.

## Provenance and peer review

Not commissioned, externally peer-reviewed.

## Acknowledgements

None.

## Supplementary Material
