# Supplementary figures and images for: Risk factors for Hirschsprung disease-associated enterocolitis: a systematic review and meta-analysis
Source: Int J Surg. 2023 Jun 5;109(8):2509–24. doi: 10.1097/JS9.0000000000000473 (PMC10442125; doi:10.1097/JS9.0000000000000473)

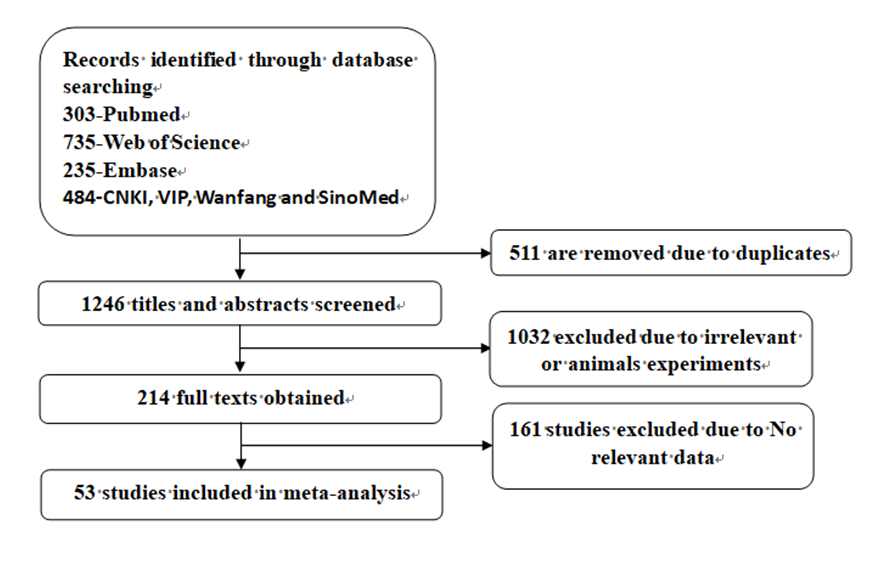

Supplement: Supplementary file 2 [file js9-109-2509-s002.tiff]
