# Supplementary material for: Risk factors for Hirschsprung disease-associated enterocolitis: a systematic review and meta-analysis
Source: Int J Surg. 2023 Jun 5;109(8):2509–24. doi: 10.1097/JS9.0000000000000473 (PMC10442125; doi:10.1097/JS9.0000000000000473)
Supplement: Supplementary file 4 [file js9-109-2509-s004.docx]

**Appendix1: search strategy**

**PUBMED:**

(((((((((((((((((((risk factor[Title/Abstract]) OR (Related factors[Title/Abstract])) OR (influence factor[Title/Abstract])) OR (predictors[Title/Abstract])) OR (Environmental factors[Title/Abstract])) OR (Gender[Title/Abstract])) OR (age[Title/Abstract])) OR (malnutrition[Title/Abstract])) OR (upper respiratory tract infection[Title/Abstract])) OR (pulmonary infection[Title/Abstract])) OR (spasm length[Title/Abstract])) OR (bowel washing time[Title/Abstract])) OR (pathological typing[Title/Abstract])) OR (surgical method[Title/Abstract])) OR (probiotics[Title/Abstract])) OR (feeding condition[Title/Abstract])) OR (surgical approach[Title/Abstract])) OR (anal dilatation[Title/Abstract])) OR (diet control[Title/Abstract])) OR (intestinal flora disturbance[Title/Abstract])

(((((Enterocolitis[Title/Abstract]) OR (coloenteritis[Title/Abstract])) OR (intestinal colitis[Title/Abstract])) OR (enteritis[Title/Abstract])) OR (enteronitis[Title/Abstract])) OR (esoenteritis[Title/Abstract])

((((Hirschsprung disease) OR (Hirschsprung's disease)) OR (HSCR)) OR (congenital megacolon[Title/Abstract])) OR (aganglionosis[Title/Abstract])

((#1) AND (#2)) AND (#3)

**Web of science**

((((TS=(Hirschsprung disease)) OR TS=(Hirschsprung's disease)) OR TS=(HSCR)) OR TS=(congenital megacolon)) OR TS=(aganglionosis)

(((((((((((((((((((TS=(risk factor)) OR TS=(Related factors)) OR TS=(influence factor)) OR TS=(predictors)) OR TS=(Environmental factors)) OR TS=(Gender)) OR TS=(age)) OR TS=(malnutrition)) OR TS=(upper respiratory tract infection)) OR TS=(pulmonary infection)) OR TS=(spasm length)) OR TS=(bowel washing time)) OR TS=( pathological typing)) OR TS=(surgical method)) OR TS=(probiotics)) OR TS=(feeding condition)) OR TS=(surgical approach)) OR TS=(anal dilatation)) OR TS=(diet control)) OR TS=( intestinal flora disturbance)

(((((TS=(Enterocolitis)) OR TS=(coloenteritis)) OR TS=(intestinal colitis)) OR TS=(enteritis)) OR TS=(enteronitis)) OR TS=(esoenteritis)

((#1) AND (#2)) AND (#3)

**Embase**

'risk factor':ti,ab,kw OR 'related factors':ti,ab,kw OR 'influence factor':ti,ab,kw OR predictors:ti,ab,kw OR 'environmental factors':ti,ab,kw OR gender:ti,ab,kw OR age:ti,ab,kw OR malnutrition:ti,ab,kw OR 'upper respiratory tract infection':ti,ab,kw OR 'pulmonary infection':ti,ab,kw OR 'spasm length':ti,ab,kw OR 'bowel washing time':ti,ab,kw OR 'pathological typing':ti,ab,kw OR 'surgical method':ti,ab,kw OR probiotics:ti,ab,kw OR 'feeding condition':ti,ab,kw OR 'surgical approach':ti,ab,kw OR 'anal dilatation':ti,ab,kw OR 'diet control':ti,ab,kw OR 'intestinal flora disturbance':ti,ab,kw

enterocolitis:ti,ab,kw OR coloenteritis:ti,ab,kw OR 'intestinal colitis':ti,ab,kw OR enteritis:ti,ab,kw OR enteronitis:ti,ab,kw OR esoenteritis:ti,ab,kw

'hirschsprung disease':ti,ab,kw OR 'hirschsprungs disease':ti,ab,kw OR hscr:ti,ab,kw OR 'congenital megacolon':ti,ab,kw OR aganglionosis:ti,ab,kw

((#1) AND (#2)) AND (#3)

中国知网：

SU='先天性巨结肠'*('小肠结肠炎'+'肠炎'+'肠结肠炎')*('危险因素'+'高危因素'+'相关因素'+'影响因素'+'预测因素'+'环境因素'+'性别'+'年龄'+'营养不良'+'上呼吸道感染'+'肺部感染'+'痉挛段长度'+'洗肠时间'+'病理分型'+'手术方式'+'益生菌'+'喂养情况'+'手术入路'+'扩肛'+'饮食控制'+'肠道菌群紊乱')

维普 2022.5.10

M=先天性巨结肠*(小肠结肠炎+肠炎+肠结肠炎)*(危险因素+高危因素+相关因素+影响因素+预测因素+环境因素+性别+年龄+营养不良+上呼吸道感染+肺部感染+痉挛段长度+洗肠时间+病理分型+手术方式+益生菌+喂养情况+手术入路+扩肛+饮食控制+肠道菌群紊乱)

中国生物医学文献库

[(((((((((("危险因素"[常用字段:智能] OR "高危因素"[常用字段:智能] OR "相关因素"[常用字段:智能] OR "影响因素"[常用字段:智能] OR "预测因素"[常用字段:智能] OR "性别"[常用字段:智能] OR "年龄"[常用字段:智能] OR "营养不良"[常用字段:智能] OR "上呼吸道感染"[常用字段:智能])) OR (("肺部感染"[常用字段:智能] OR "痉挛段长度"[常用字段:智能] OR "洗肠时间"[常用字段:智能] OR "病理分型"[常用字段:智能] OR "手术方式"[常用字段:智能] OR "益生菌"[常用字段:智能] OR "喂养情况"[常用字段:智能] OR "手术入路"[常用字段:智能] OR "扩肛"[常用字段:智能])))) OR (("饮食控制"[常用字段:智能] OR "肠道菌群紊乱"[常用字段:智能] OR "环境因素"[常用字段:智能]))))) AND (("小肠结肠炎"[常用字段:智能] OR "肠炎"[常用字段:智能] OR "肠结肠炎"[常用字段:智能])))) AND (先天性巨结肠))](javascript:toDoRelimitSearch();)

万方

题名或关键词:“先天性巨结肠”and(“小肠结肠炎”or“肠炎”or“肠结肠炎”)and(“危险因素”or“高危因素”or“相关因素”or“影响因素”or“预测因素”or“环境因素”or“性别”or“年龄”or“营养不良”or“上呼吸道感染”or“肺部感染”or“痉挛段长度”or“洗肠时间”or“病理分型”or“手术方式”or“益生菌”or“喂养情况”or“手术入路”or“扩肛”or“饮食控制”or“肠道菌群紊乱”)

**AppendixⅡ：Forest plots from case-control studies or cohort studies analyzed separately.**


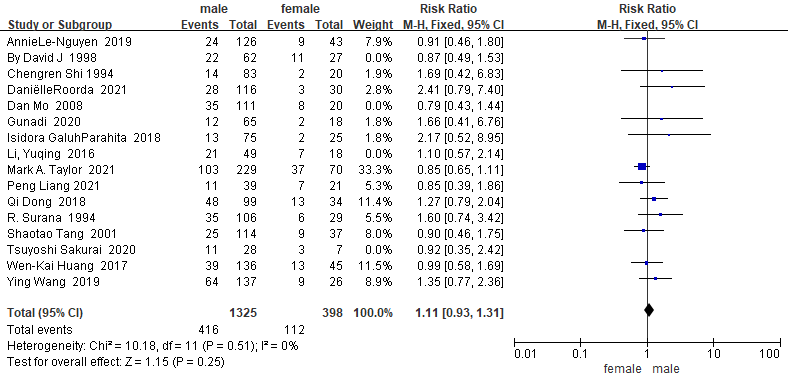


Appendix figure 1 Forest plot of gender for postoperative HAEC (cohort study)


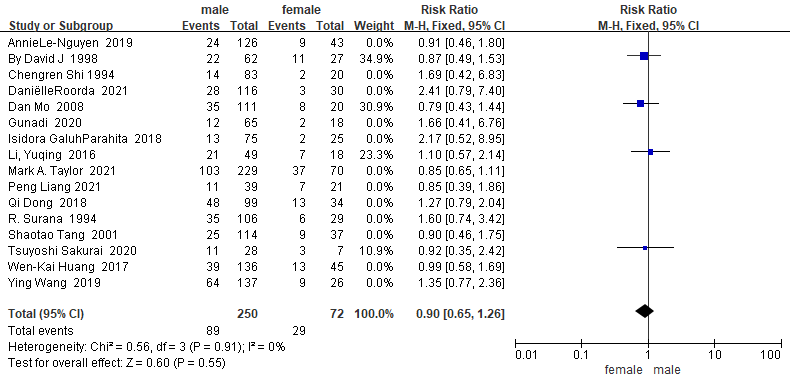


Appendix figure 2 Forest plot of gender for postoperative HAEC (case-control study)


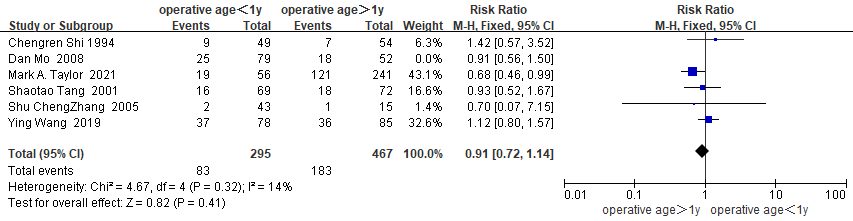


Appendix figure 3 Forest plot of operation age >1 year for postoperative HAEC (cohort study)


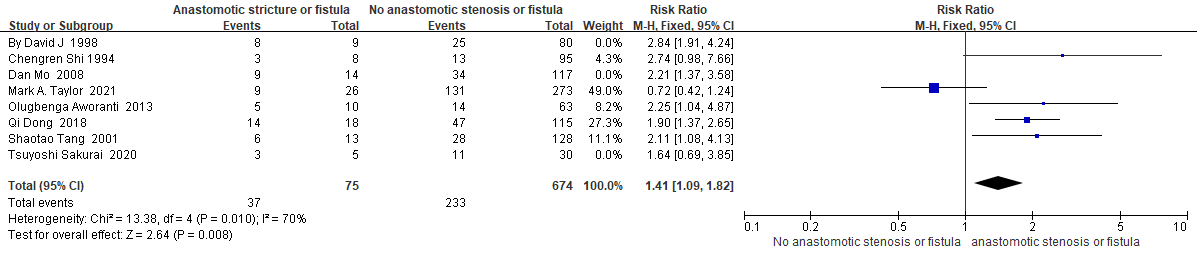


Appendix figure 4 Forest plot of anastomotic stenosis or fistula for postoperative HAEC (cohort study)


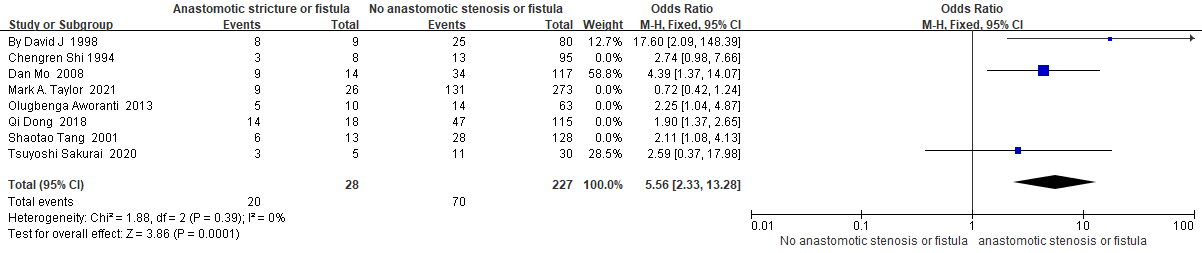


Appendix figure 5 Forest plot of anastomotic stenosis or fistula for postoperative HAEC (case-control study)


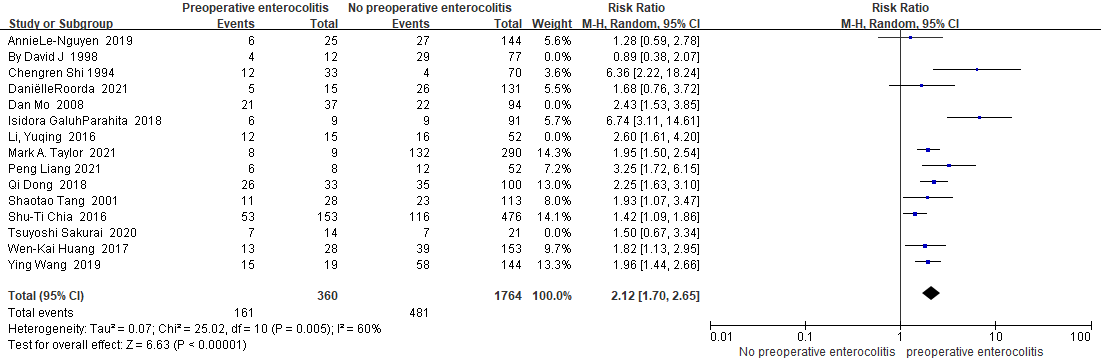


Appendix figure 6 Forest plot of preoperative enterocolitis for postoperative HAEC (cohort study)


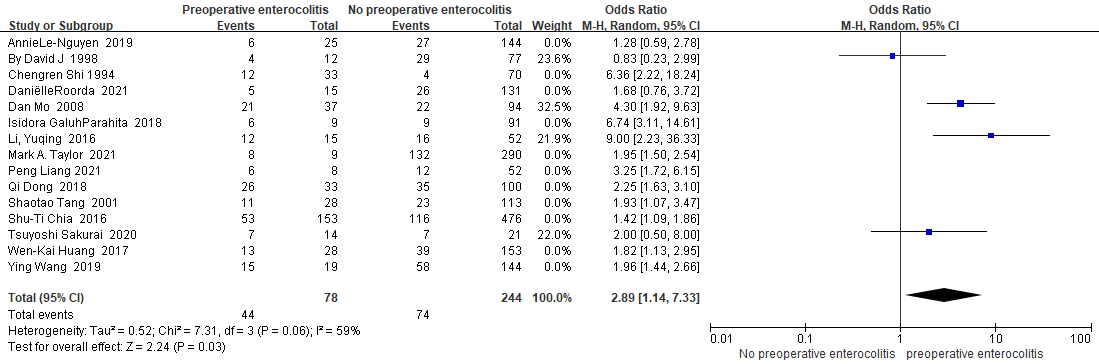


Appendix figure 7 Forest plot of preoperative enterocolitis for postoperative HAEC (case-control study)


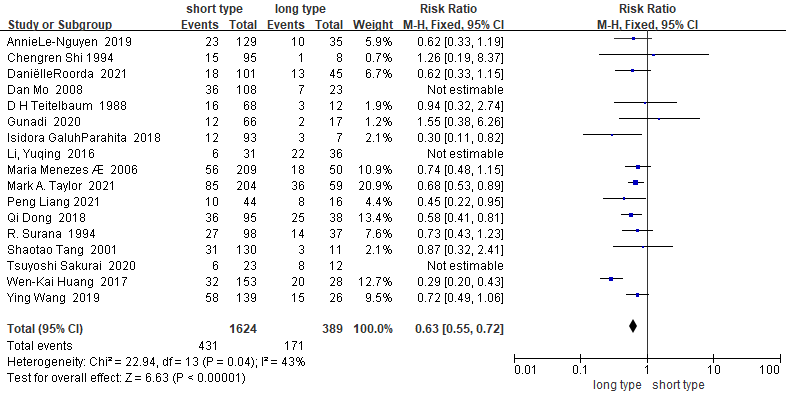


Appendix figure 8 Forest plot of pathological type for postoperative HAEC (cohort study)


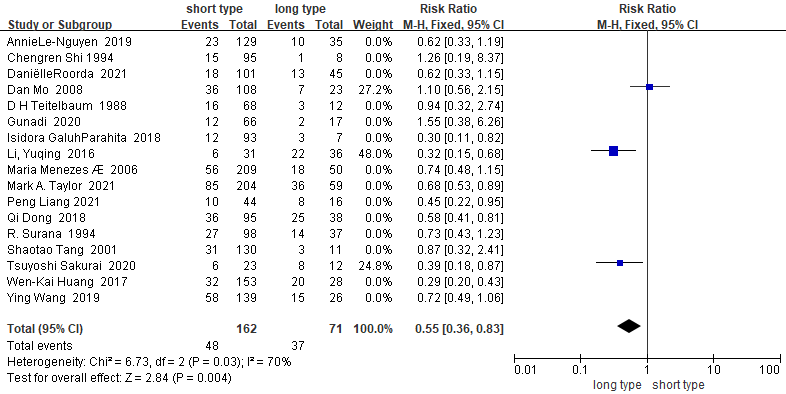


Appendix figure 9 Forest plot of pathological type for postoperative HAEC (case-control study)


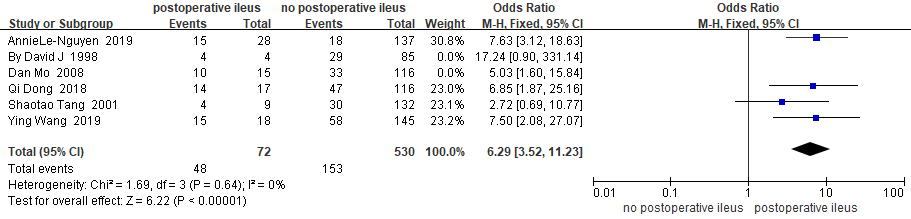


Appendix figure 10 Forest plot of postoperative ileus for postoperative HAEC (cohort study)


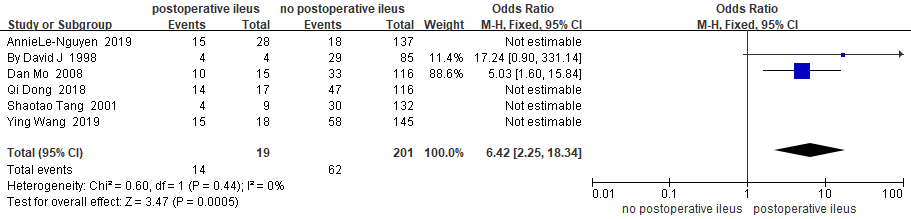


Appendix figure 11 Forest plot of postoperative ileus for postoperative HAEC (case-control study)


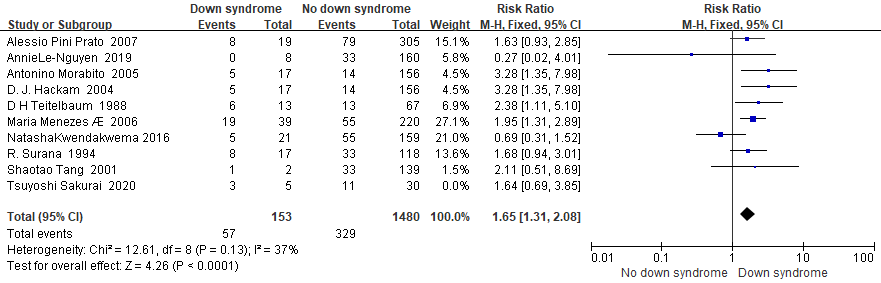


Appendix figure 12 Forest plot of Down syndrome for postoperative HAEC (cohort study)


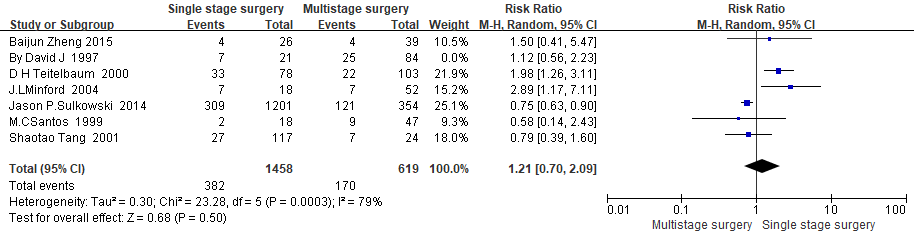


Appendix figure 13 Forest plot of staging operation for postoperative HAEC (cohort study)

**AppendixⅢ: The forest(More than 3 studies included) of anal dilatation, staging operation, operation age, Down syndrome, surgical age for preoperative enterocolitis, comparison of Soave and Duhamel, comparison of Soave and Swenson, comparison of Duhamel and TEPT, comparison of Duhamel and Swenson, comparison of laparotomy and laparoscope. (Details of the forest plots for the non-statistically significant factors)**

**
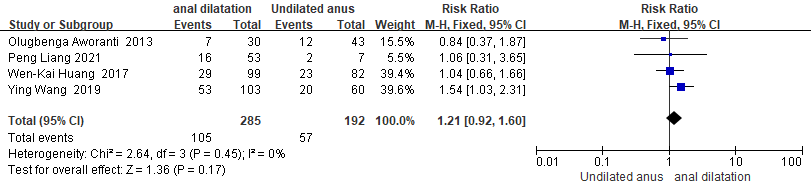
**

Appendix figure 14 The forest of anal dilatation.

**
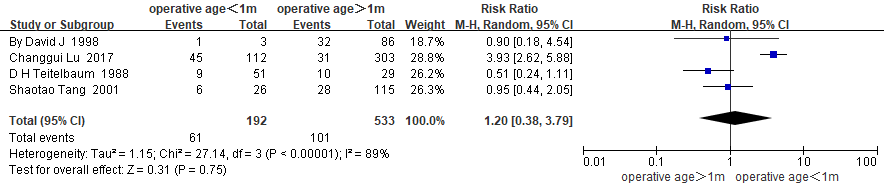
**

Appendix figure 15 The forest of operation age >1 month.

**
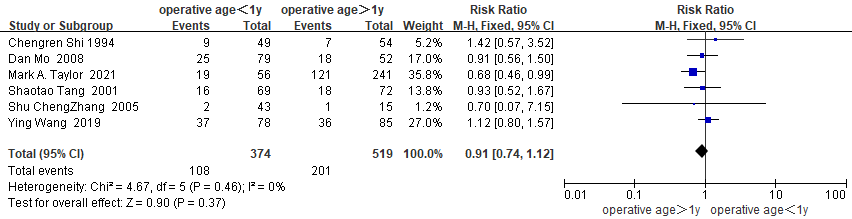
**

Appendix figure 16 The forest of operation age >1 year.

**
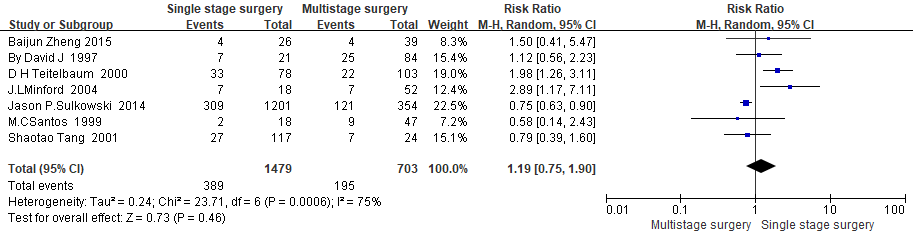
**

Appendix figure 17 The forest of staging operation.

**
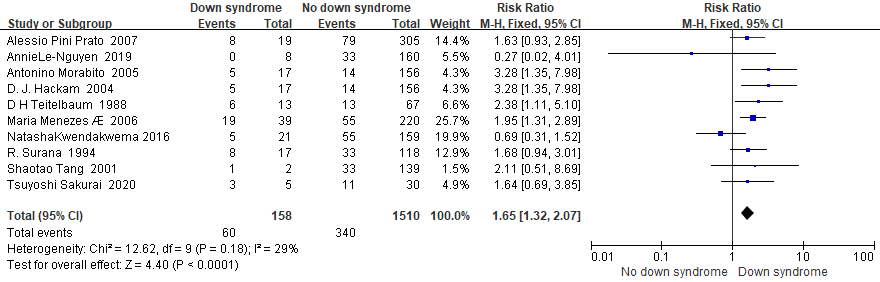
**

Appendix figure 18 The forest of Down syndrome.

**
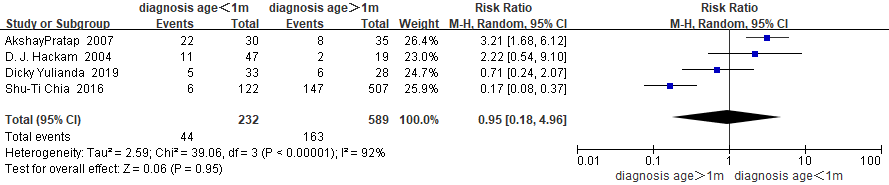
**

Appendix figure 19 The forest of surgical age for preoperative enterocolitis.

**
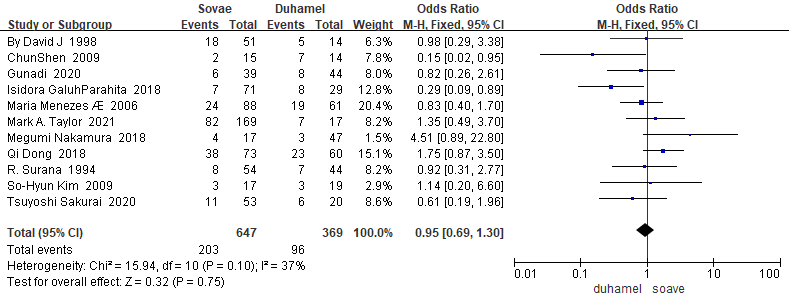
**

Appendix figure 20 The forest of comparison of Soave and Duhamel.

**
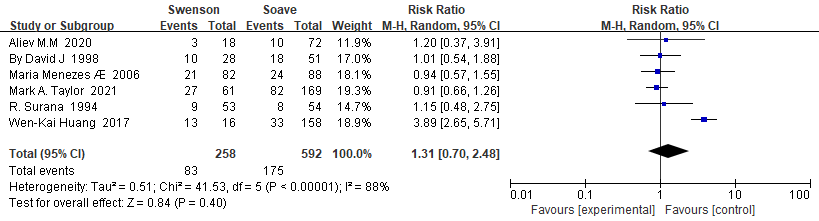
**

Appendix figure 21 The forest of comparison of Soave and Swenson.

**
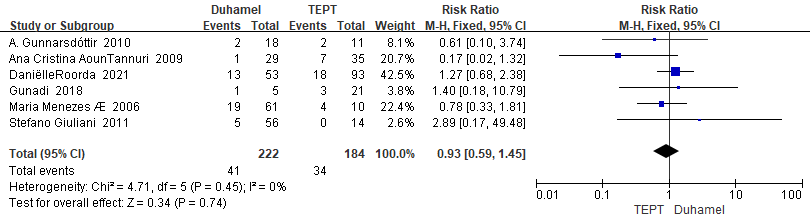
**

Appendix figure 22 The forest of comparison of Duhamel and TEPT.

**
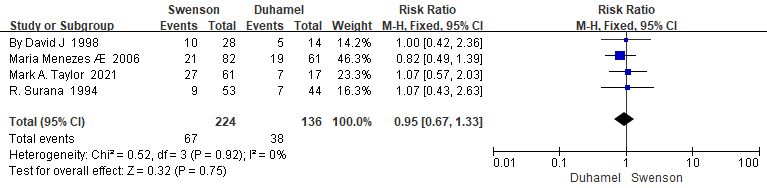
**

Appendix figure 23 The forest of comparison of Duhamel and Swenson.

**
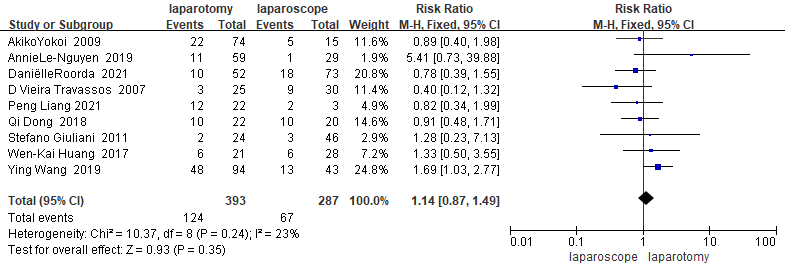
**

Appendix figure 24 The forest of comparison of laparotomy and laparoscope.

**AppendixⅣ： The sensitivity analysis and bais of preoperative malnutrition, preoperative respiratory infection or pneumonia, preoperative hypoproteinemia surgical access, anastomotic stricture or fistula, preoperative enterocolitis, pathological type, postoperative ileus, Down syndrome, length of ganglionless segment and the funnel plot of all the factors (More than 3 studies included) we analyzed.**

**
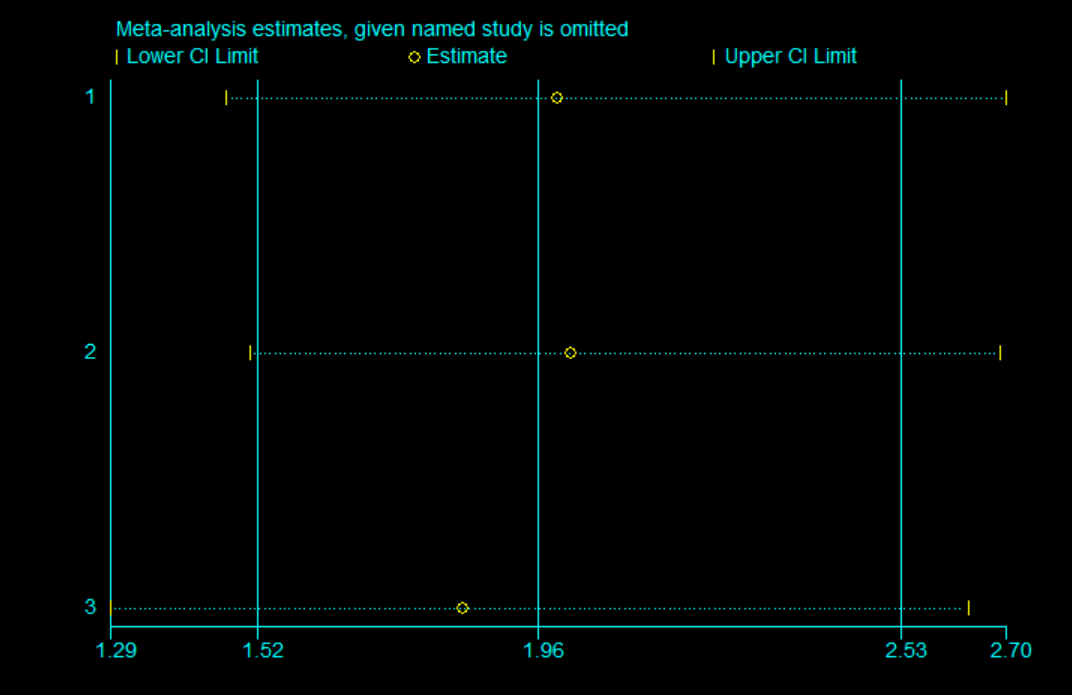
**

Appendix figure 25 The sensitivity of preoperative malnutrition.

**
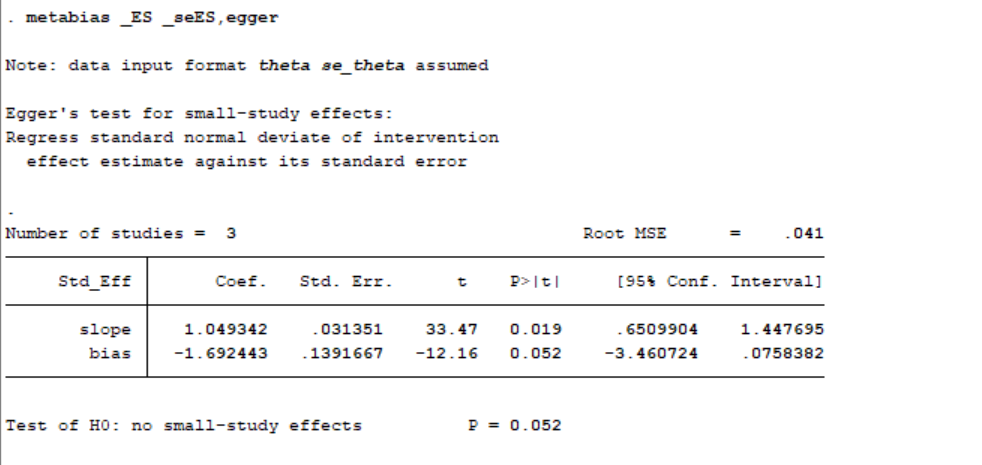
**

Appendix figure 26 The bias of preoperative malnutrition.

**
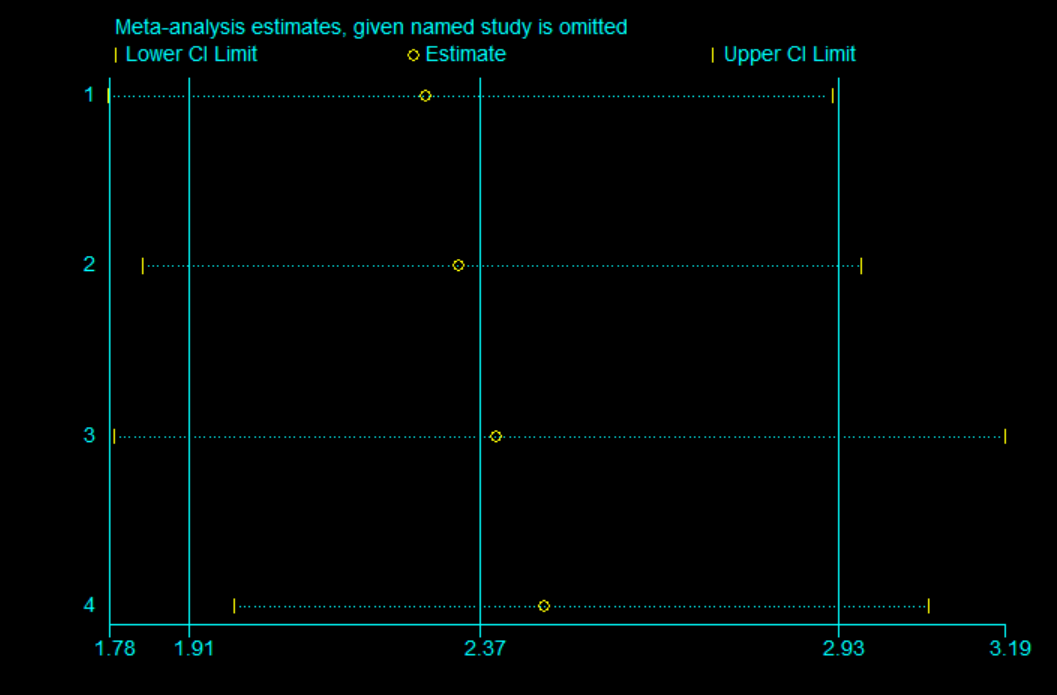
**

Appendix figure 27 The sensitivity of preoperative respiratory infection or pneumonia. **
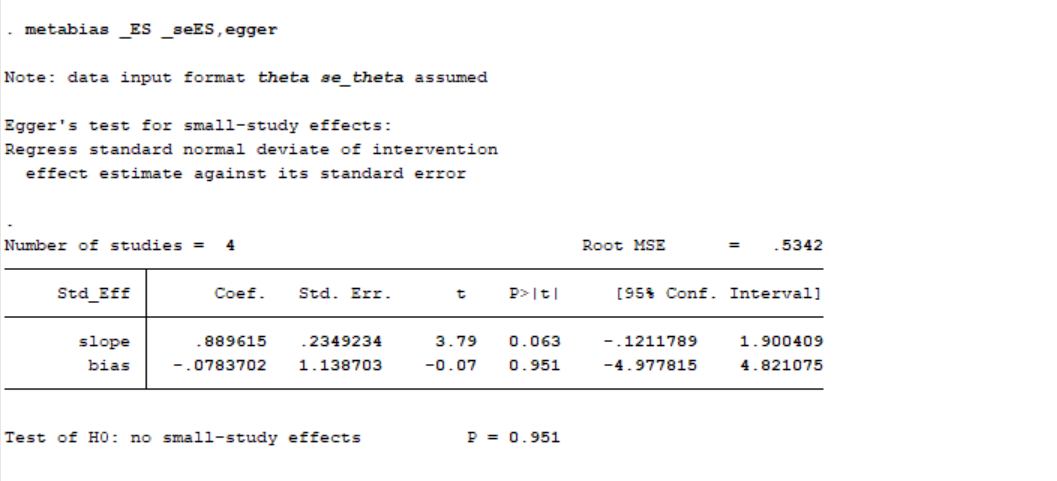
**

Appendix figure 28 The bias of preoperative respiratory infection or pneumonia.

**
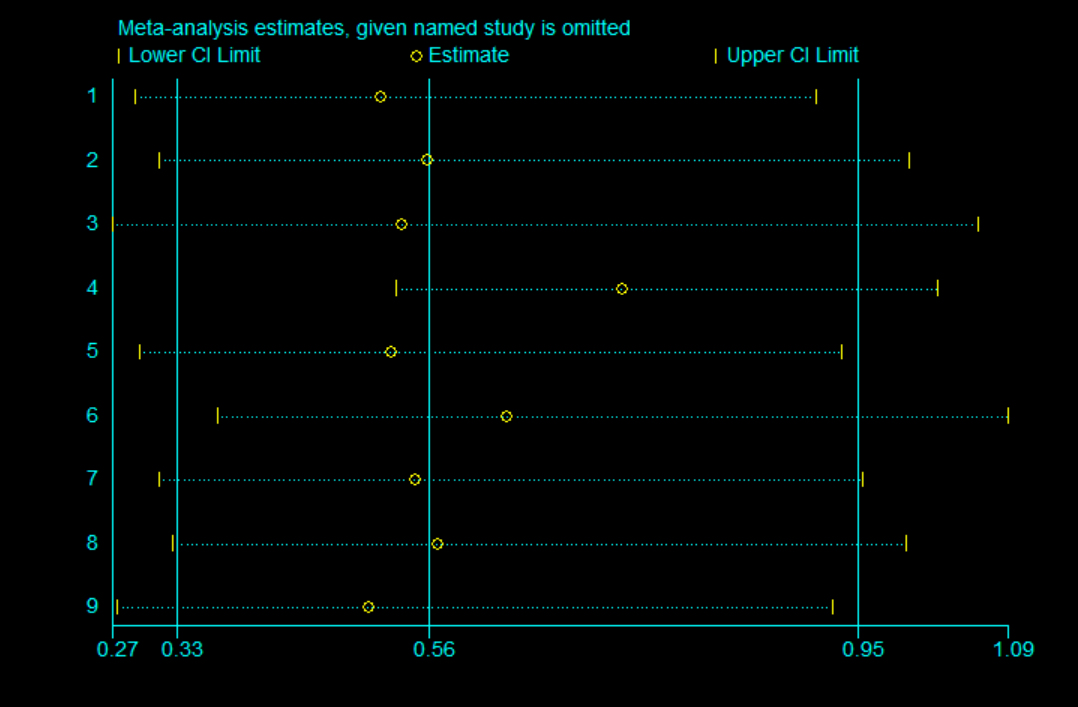
**

Appendix figure 29 The sensitivity of surgical access.

**
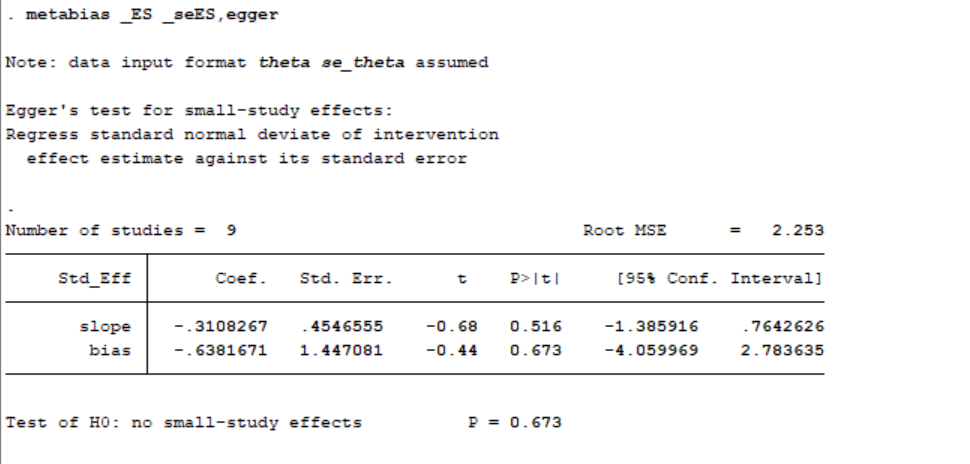
**

Appendix figure 30 The bias of surgical access.

**
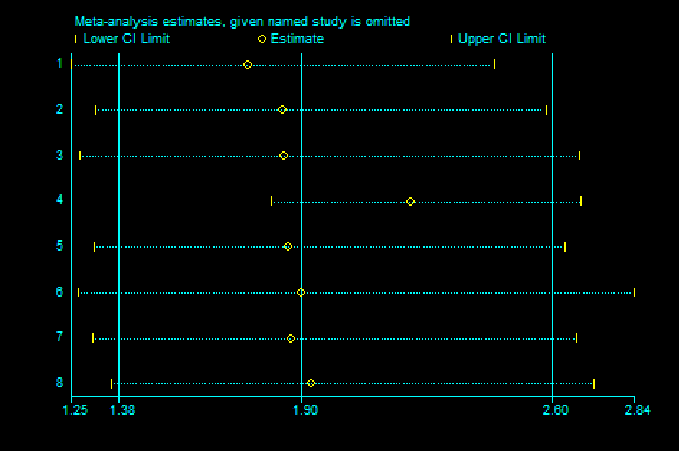
**

Appendix figure 31 The sensitivity of anastomotic stricture or fistula.

**
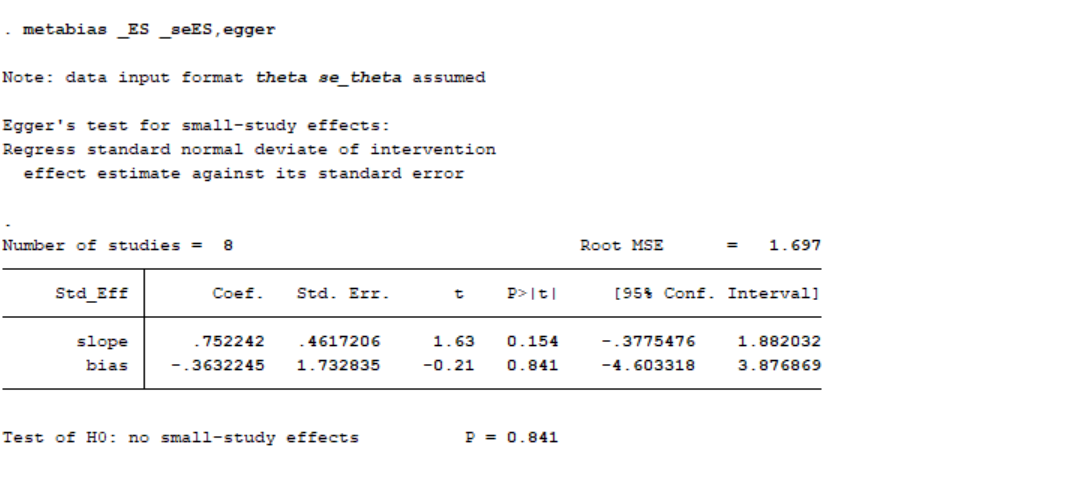
**

Appendix figure 32 The bias of anastomotic stricture or fistula.

**
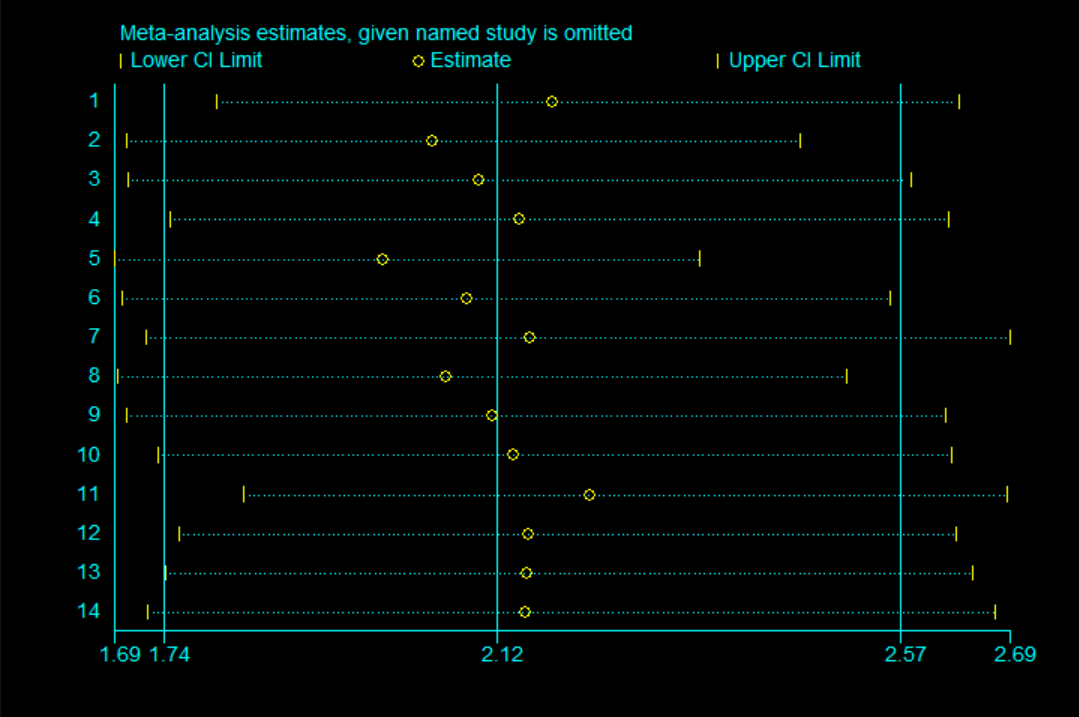
**

Appendix figure 33 The sensitivity of preoperative enterocolitis.

**
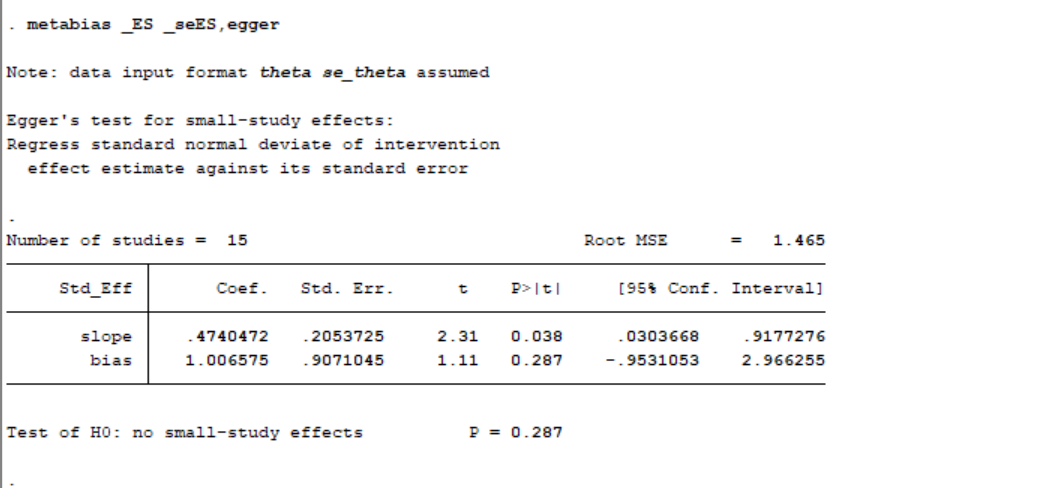
**

Appendix figure 34 The bias of preoperative enterocolitis.

**
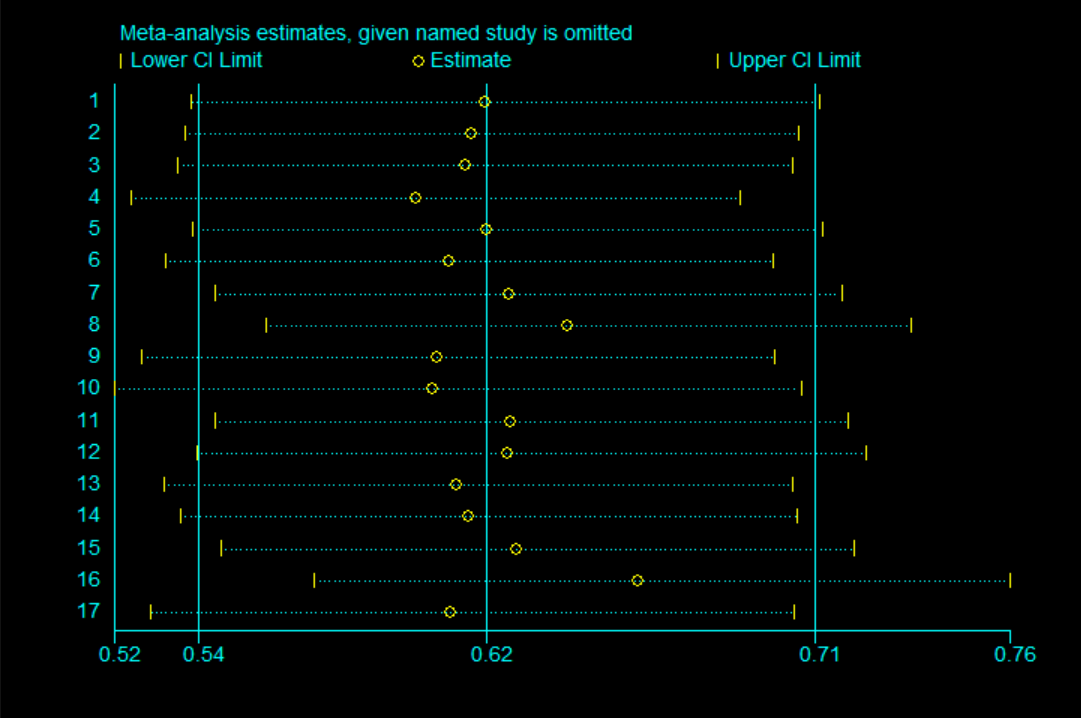
**

Appendix figure 35 The sensitivity of pathological type.

**
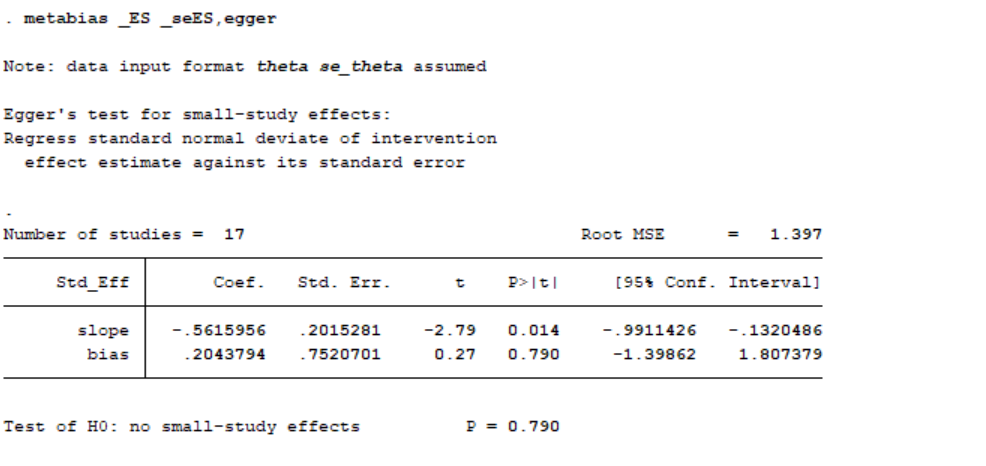
**

Appendix figure 36 The bias of pathological type.

**
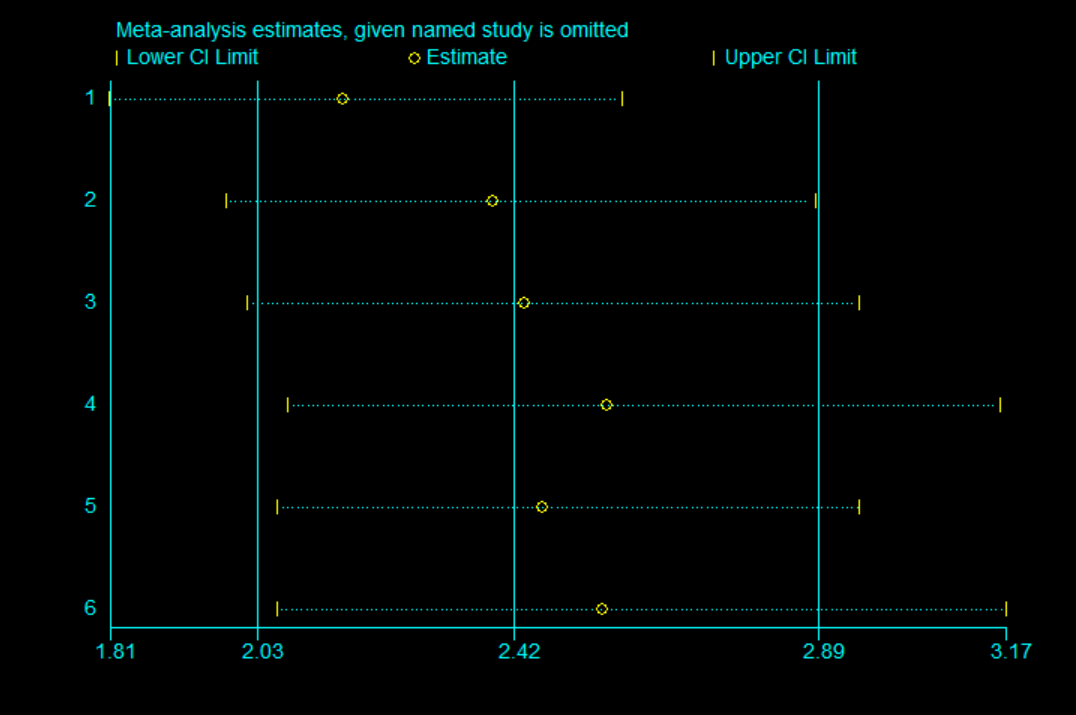
**

Appendix figure 37 The sensitivity of postoperative ileus.

**
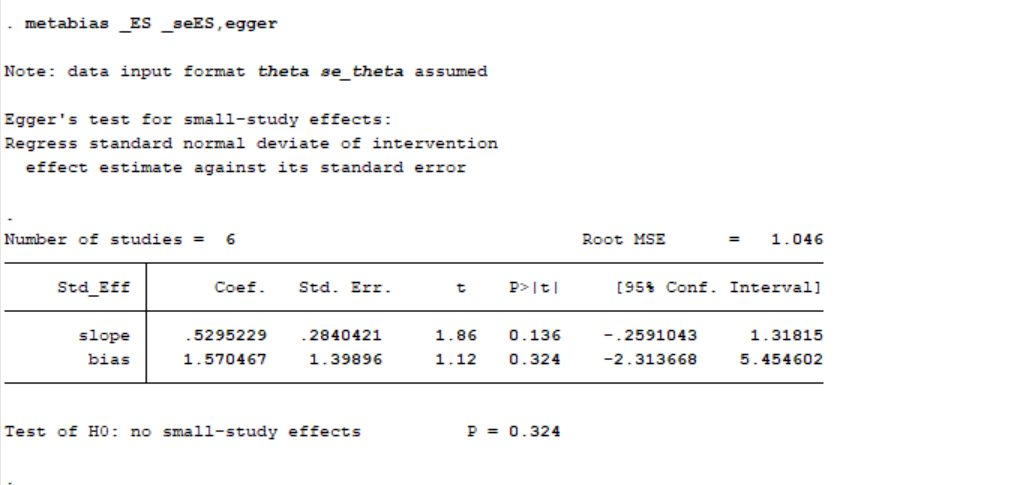
**

Appendix figure 38 The bias of postoperative ileus.

**
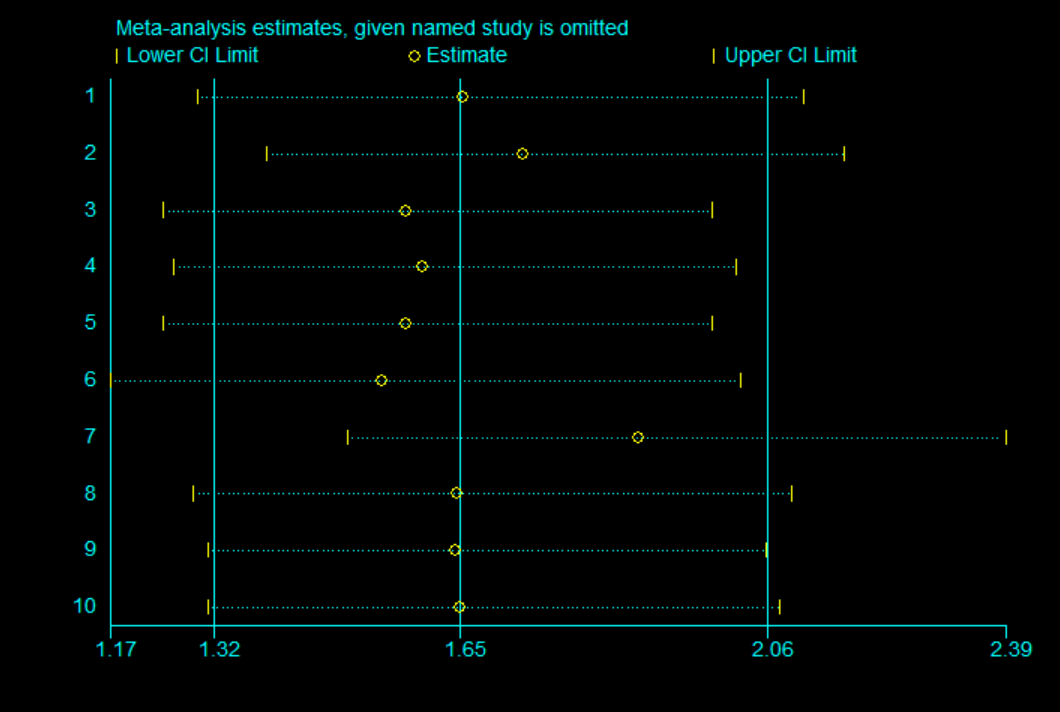
**

Appendix figure 39 The sensitivity of length of Down syndrome.

**
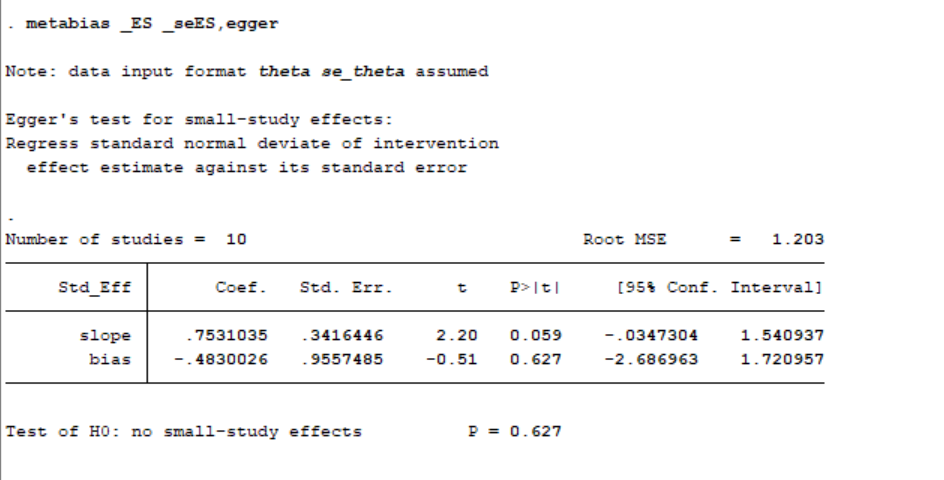
**

Appendix figure 40 The bias of length of Down syndrome.

**
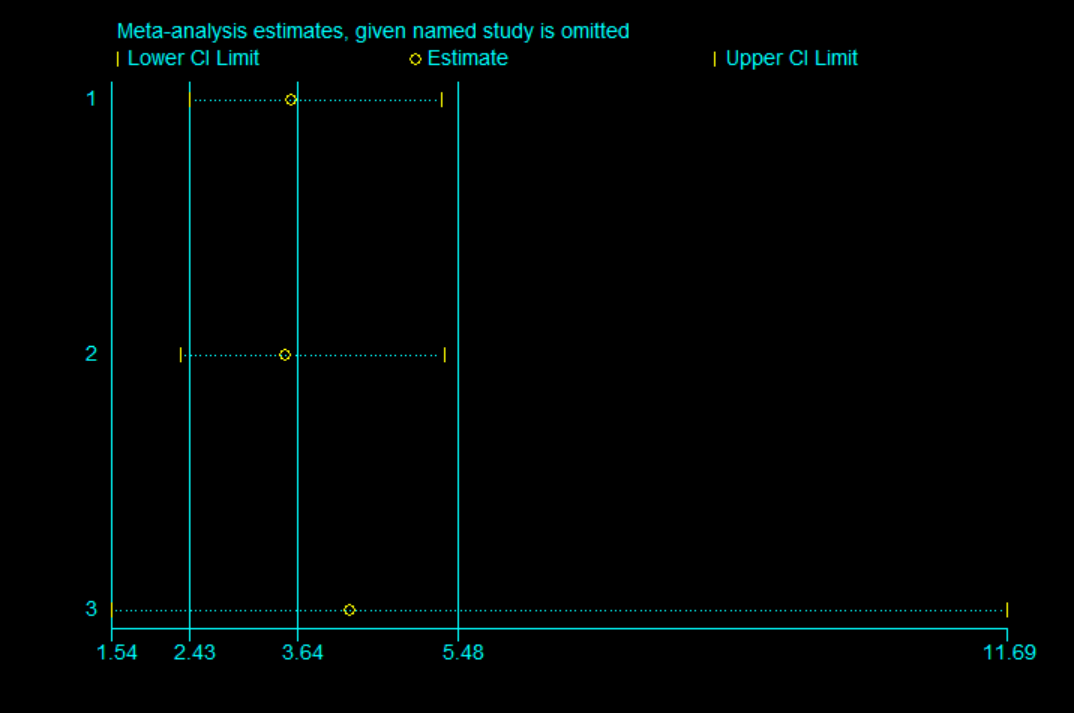
**

Appendix figure 41 The sensitivity of length of ganglionless segment >30 cm.

**
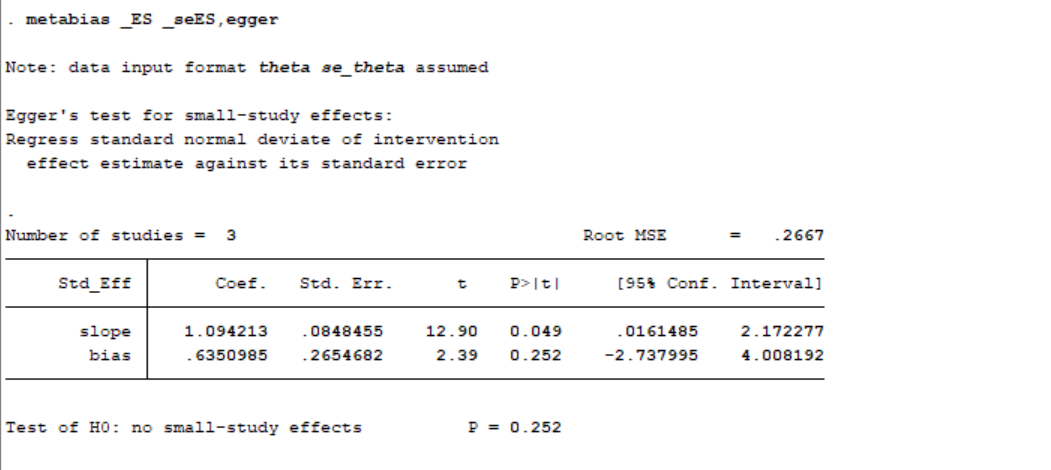
**

Appendix figure 42 The bias of length of ganglionless segment >30 cm.

**
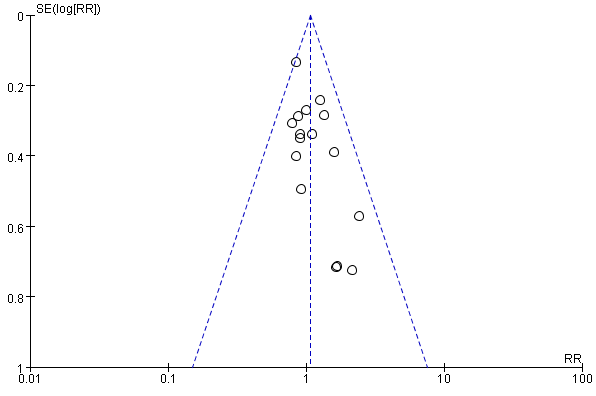
**

Appendix figure 43 The funnel plot of gender.

**
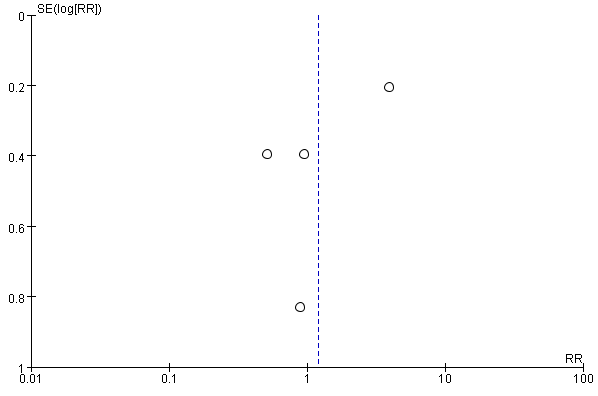
**

Appendix figure 44 The funnel plot of operative age >1 month.

**
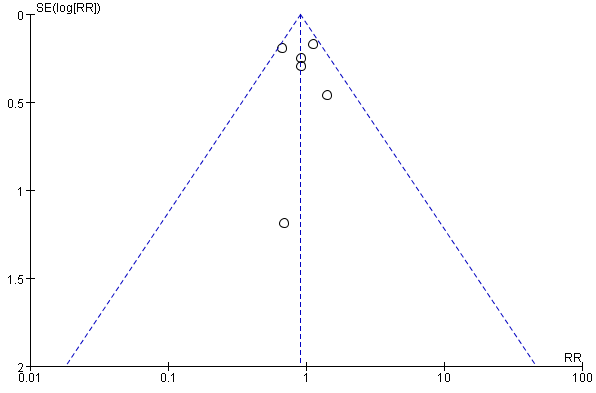
**

Appendix figure 45 The funnel plot of operative age >1 year.

**
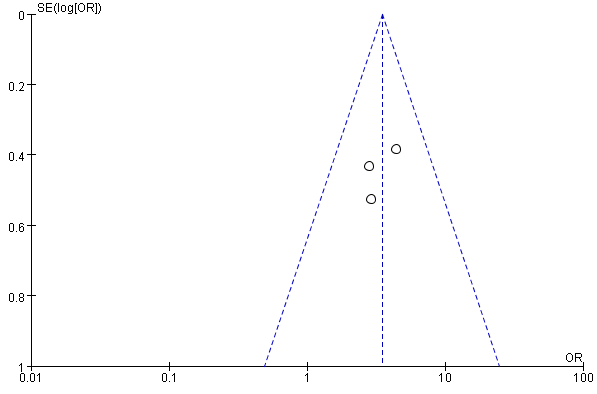
**

Appendix figure 46 The funnel plot of preoperative malnutrition.

**
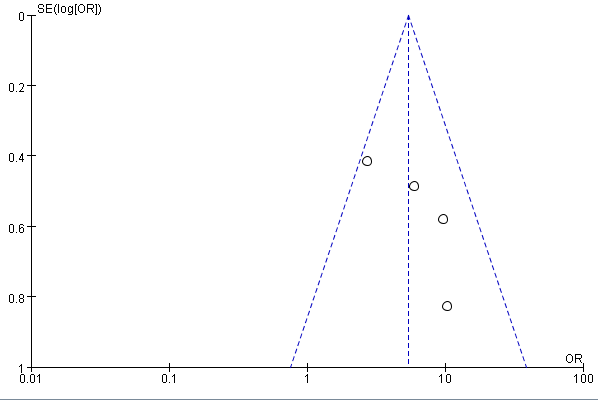
**

Appendix figure 47 The funnel plot of Preoperative respiratory infection or pneumonia.

**
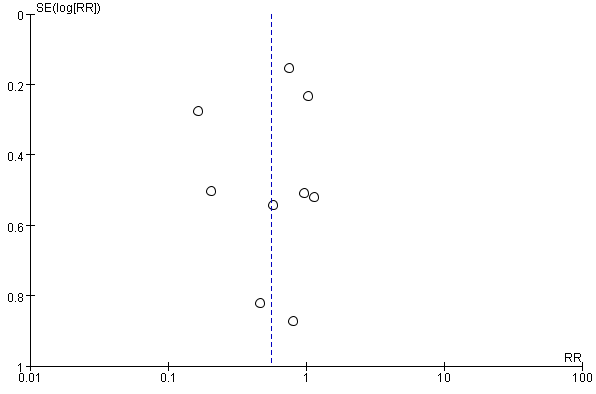
**

Appendix figure 48 The funnel plot of comparison of transanal surgery and transabdominal.

**
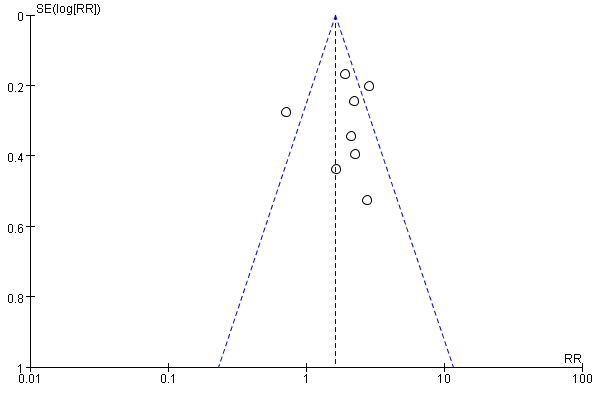
**

Appendix figure 49 The funnel plot of anastomotic stricture or fistula.

**
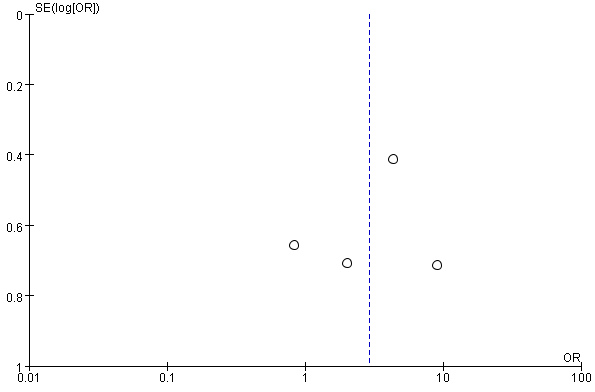
**

Appendix figure 50 The funnel plot of Preoperative enterocolitis.

**
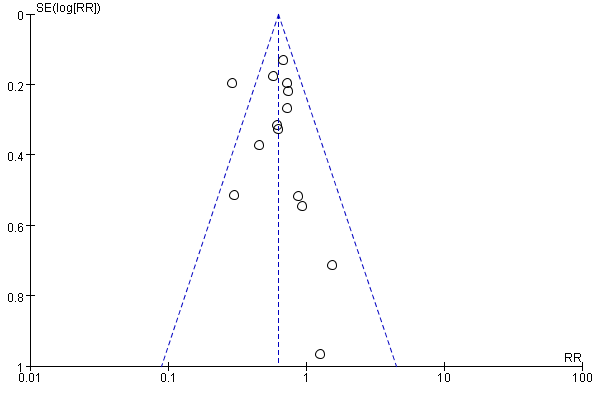
**

Appendix figure 51 The funnel plot of pathological type.

**
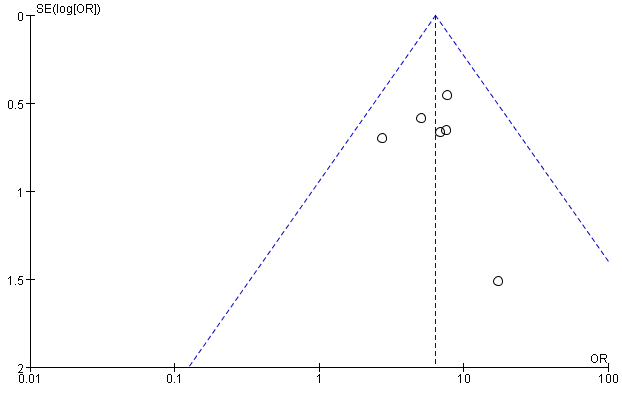
**

Appendix figure 52 The funnel plot of postoperative ileus.

**
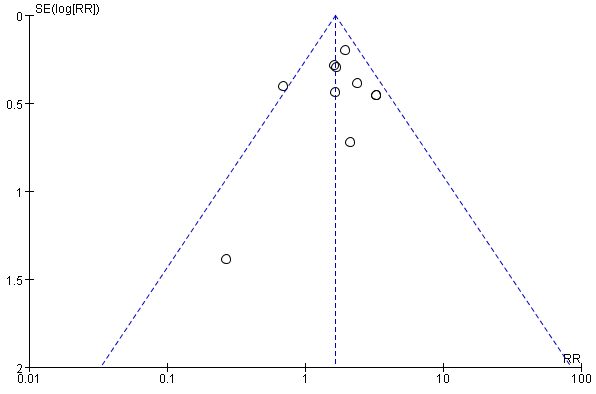
**

Appendix figure 53 The funnel plot of Down syndrome.

**
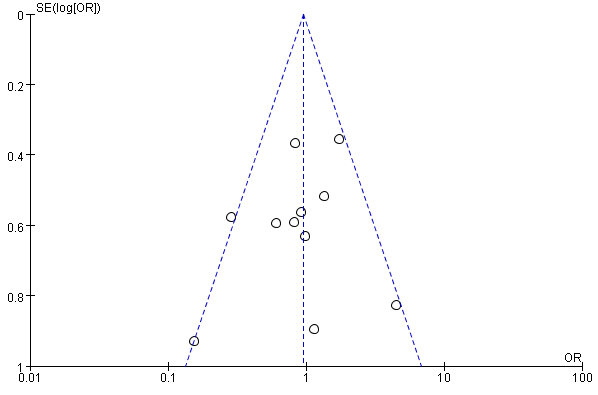
**

Appendix figure 54 The funnel plot of comparison of soave and Duhamel.

**
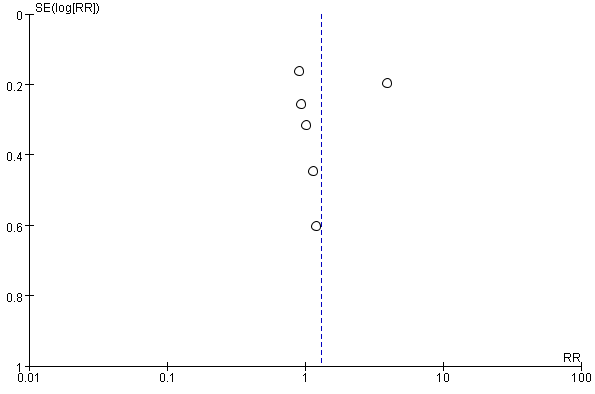
**

Appendix figure 55 The funnel plot of comparison of soave and Swenson.

**
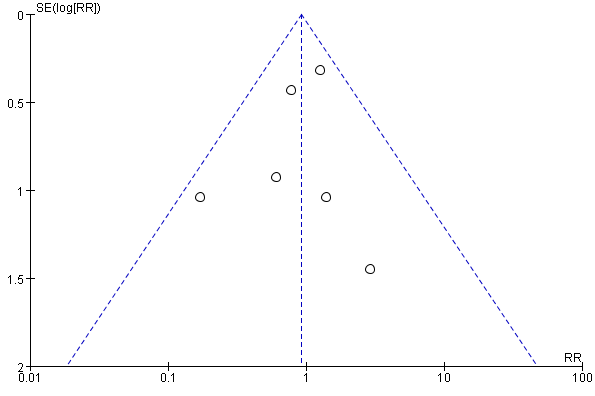
**

Appendix figure 56 The funnel plot of comparison of Duhamel and TEPT.

**
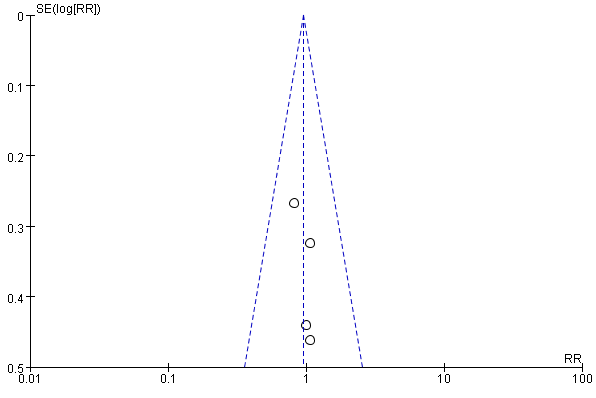
**

Appendix figure 57 The funnel plot of comparison of Swenson and Duhamel.

**
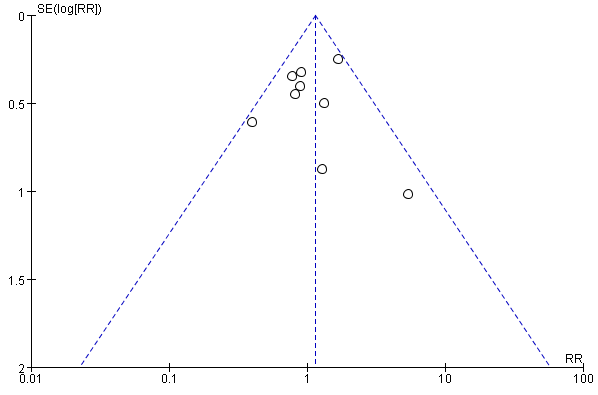
**

Appendix figure 58 The funnel plot of comparison of laparotomy and laparoscope.

**
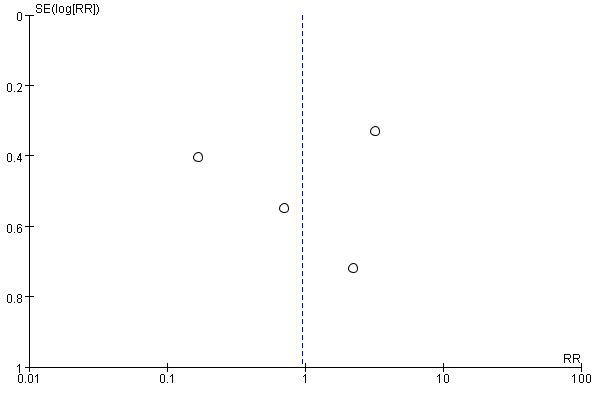
**

Appendix figure 59 The funnel plot of diagnosis age for preoperative HAEC.

**
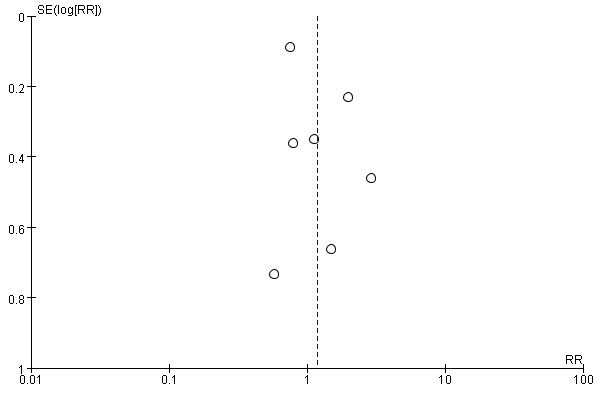
**

Appendix figure 60 The funnel plot of staging operation.

**
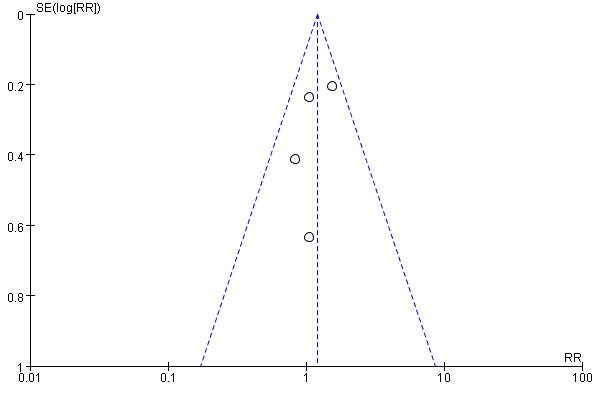
**

Appendix figure 61 The funnel plot of anal dilatation.
